# Scientometrics Evaluation of Published Scientific Papers on the Use of Proteomics Technologies in Mastitis Research in Ruminants

**DOI:** 10.3390/pathogens13040324

**Published:** 2024-04-15

**Authors:** Maria V. Bourganou, Dimitris C. Chatzopoulos, Daphne T. Lianou, George Th. Tsangaris, George C. Fthenakis, Angeliki I. Katsafadou

**Affiliations:** 1 Faculty of Public and One Health, University of Thessaly, 43100 Karditsa, Greece; mbourganou@uth.gr (M.V.B.); dchatzopoulos@uth.gr (D.C.C.); 2 Veterinary Faculty, University of Thessaly, 43100 Karditsa, Greece; dlianou@vet.uth.gr (D.T.L.);; 3 Proteomics Research Unit, Biomedical Research Foundation of the Academy of Athens, 11527 Athens, Greece; gthtsangaris@bioacademy.gr

**Keywords:** bibliometric analysis, cattle, goat, LC/MS-MS, mammary infection, mastitis, meta-research, One Health, proteome, proteomics, sheep, staphylococcus, subclinical mastitis

## Abstract

The objective of this study was the presentation of quantitative characteristics regarding the scientific content and bibliometric details of the relevant publications. In total, 156 papers were considered. Most papers presented original studies (*n* = 135), and fewer were reviews (*n* = 21). Most original articles (*n* = 101) referred to work involving cattle. Most original articles described work related to the diagnosis (*n* = 72) or pathogenesis (*n* = 62) of mastitis. Most original articles included field work (*n* = 75), whilst fewer included experimental (*n* = 31) or laboratory (*n* = 30) work. The tissue assessed most frequently in the studies was milk (*n* = 59). Milk was assessed more frequently in studies on the diagnosis (61.1% of relevant studies) or pathogenesis (30.6%) of the infection, but mammary tissue was assessed more frequently in studies on the treatment (31.0%). In total, 47 pathogens were included in the studies described; most were Gram-positive bacteria (*n* = 34). The three bacteria most frequently included in the studies were *Staphylococcus aureus* (*n* = 55 articles), *Escherichia coli* (*n* = 31) and *Streptococcus uberis* (*n* = 19). The proteomics technology employed more often in the respective studies was liquid chromatography-tandem mass spectrometry (LC-MS/MS), either on its own (*n* = 56) or in combination with other technologies (*n* = 40). The median year of publication of articles involving bioinformatics or LC-MS/MS and bioinformatics was the most recent: 2022. The 156 papers were published in 78 different journals, most frequently in the *Journal of Proteomics* (*n* = 16 papers) and the *Journal of Dairy Science* (*n* = 12). The median number of cited references in the papers was 48. In the papers, there were 1143 co-authors (mean: 7.3 ± 0.3 co-authors per paper, median: 7, min.–max.: 1–19) and 742 individual authors. Among them, 15 authors had published at least seven papers (max.: 10). Further, there were 218 individual authors who were the first or last authors in the papers. Most papers were submitted for open access (*n* = 79). The median number of citations received by the 156 papers was 12 (min.–max.: 0–339), and the median yearly number of citations was 2.0 (min.–max.: 0.0–29.5). The *h*-index of the papers was 33, and the *m*-index was 2. The increased number of cited references in papers and international collaboration in the respective study were the variables associated with most citations to published papers. This is the first ever scientometrics evaluation of proteomics studies, the results of which highlighted the characteristics of published papers on mastitis and proteomics. The use of proteomics in mastitis research has focused on the elucidation of pathogenesis and diagnosis of the infection; LC-MS/MS has been established as the most frequently used proteomics technology, although the use of bioinformatics has also emerged recently as a useful tool.

## 1. Introduction

Mastitis is the inflammation of the mammary gland, caused by the invasion of bacteria into the mammary parenchyma. The infection is characterized by increased cell content in the mammary parenchyma and milk, coupled with bacterial shedding in milk. Clinical features include systemic signs (e.g., fever) and mammary signs. The latter refer to the enlargement of the affected mammary gland, which becomes hot and painful, skin discoloration, hardness of the gland or the development of nodules therein; further, prominent changes also occur in the mammary secretion, which becomes clotted, serous or hemorrhagic. The infection reduces the welfare of affected animals. Moreover, in dairy animals, the infection has paramount financial importance, as its primary consequences relate to reduced milk production and the downgrading of milk quality. Further, mastitis is significant within the One Health context [1,2], first because the infection adversely influences the health and welfare of affected animals and second because there is also a potential for human transmission of the bacteria that are shed in the milk of affected animals; more important, however, is the possibility for the transfer of antibiotic resistance genes through the milk produced for human consumption [1,2,3].

The objective of proteomics work is the identification of proteins of interest within a sample and the recognition and quantification of changes in protein expression as the result of pathological conditions. Several technological approaches can be employed in order to achieve these, and they may depend upon the type of sample and the equipment available. Proteomics can be applied as bottom-up or top-down approaches [4,5]. In the bottom-up methodologies of protein identification, the basic process is that the protein mixture is digested into a peptide mixture with or without separation and subsequently introduced into the mass spectrometer for identification. In the top-down approaches, intact proteins are first separated from complex biological samples and then ionized directly by electrospray ionization (ESI) or matrix-assisted laser desorption/ionization (MALDI) technology. In both approaches, proteins or complex protein mixtures can be separated by gel-dependent (e.g., two-dimensional gel electrophoresis (2-DE)) or gel-independent (e.g., liquid chromatography (LC)) techniques prior to the introduction into the mass spectrometer for identification.

Scientometrics refers to the study of measuring and analyzing scientific literature. It reveals new data by employing information from previously published papers and aims to produce a quantitative evaluation of the initial research papers under study.

There have been very few studies on veterinary scientometrics internationally. An investigation on the Web of Science platform by using the search terms [‘scientometrics’ AND (‘veterinary’ OR ‘animal’)] revealed only 17 published papers by the end of 2023. Among these, Gupta et al. [6] studied the international research output on camels (3089 articles from 2003 to 2012), Gonzalez and Salgado-Arroyo [7] studied the research output related to veterinary work in Colombia (3000 articles from 2010 to 2019), Vaziri et al. [8] discussed the papers dealing with poultry during the 100 years up to 2022 (22,451 articles), Lianou and Fthenakis [9] analyzed papers on sheep and goats from Greece (1080 articles up to 2022), and Ding et al. [10] performed a scientometrics study of meta-analysis in the agricultural sciences (2226 articles from 1992 to 2021).

The present study is a scientometrics evaluation of published papers on proteomics as employed in mastitis research. The objective of the study was the presentation of quantitative characteristics regarding the scientific content and bibliometric details of the relevant publications.

## 2. Materials and Methods

### 2.1. Search Procedure

The Web of Science platform (www.webofknowledge.com; Clarivate Analytics, London, UK) was used for the search of relevant publications. For the search, we used the Web of Science Core Collection, in a search that spanned across multiple disciplines; the platform includes the Emerging Sources Citation Index, the Science Citation Index Expanded, the Arts and Humanities Citation Index, the Social Sciences Citation Index, the Book Citation Index and the Conference Proceedings Citation Index.

A topic search using the following terms was carried out: [[mastitis OR *mammary infection*] AND proteom*]; in this string, the asterisk served as a truncation symbol to include variations of the terms (e.g., proteome or proteomics). A topic search retrieved records that included the query terms in the title, keywords or abstract. The search was performed on 3 February 2024 (‘freeze date’). Only records published up to the 31 December 2023 were included in the study.

The initial search produced 229 records. Thereafter, document analysis of these records was performed, during which only the following types of documents were included: ‘article’ and ‘review article’. Thus, 220 papers were retained for further assessment individually.

### 2.2. Paper Evaluation

During the evaluation of the above papers, those not including work related to mastitis or proteomics were excluded from further evaluation. Finally, 156 papers remained and were included in the scientometrics evaluation. In each paper, the following details were recorded:

- Year of publication of paper.
- Country and scientific establishment (university or other institution) of origin of the paper (the country(ies) and the establishment(s) only of the first/last authors were taken into account). If multiple authors were listed as first or last authors in the papers, they were all considered.
- Type of paper: (i) original article or (ii) review. For original articles, the following details were further recorded:
  ▪ Mammalian species involved in the study described.
  ▪ Mastitis aspect(s) described therein: (i) aetiology, (ii) pathogenesis, (iii) diagnosis, (iv) treatment.
  ▪ Type of study described therein: (i) experimental work (i.e., challenge-associated), (ii) field work or (iii) laboratory work.
  ▪ Material assessed by means of the proteomics technologies employed in the study described; this included (i) blood, (ii) mammary tissue(s), (iii) milk, (iv) milk fat globule, (v) saliva or (vi) pathogen(s).
  ▪ Methodological approaches for proteomics analyses employed in the study described.
  ▪ Use of additional -omics technologies in the study described.
- Journal in which paper was published.
- Number of literature references included in the relevant list.
- Number and names of all co-authors in paper.
- Total number of citations received by the paper until the end of 2023.
- Accessibility of paper, i.e., whether there was open or subscription-only access to the paper.

### 2.3. Data Management and Analysis

The number of papers published on [mastitis OR *mammary infection*] only and [proteom*] only was also assessed by using the same procedure, in order to compare with the number of papers published on the topic of the current study.

For assessment of the impact of papers published, the following bibliometrics measures were employed: total number of citations received, *h*-index and *m*-index. The number of citations received by papers was normalized for the year of publication by calculating the average citations per year after publication of each paper.

All data were entered into Microsoft Excel and analyzed using SPSS v. 21 (IBM Analytics, Armonk, NY, USA). Descriptive analysis was performed initially. The frequency of the various outcomes was evaluated in tables of cross-categorized frequency data by use of Pearson chi-square test or Fisher exact test, as appropriate. Comparisons of proportions were performed by a two-proportion z-test. Comparisons between continuous data were performed by use of Mann–Whitney test or Kruskall–Wallis test. Linear regression analysis was used to establish associations with the year of publication of each paper. Spearman rank correlation analysis was performed as indicated, and significance of the result was evaluated according to the critical values for *r*. Associations of proteomics methodological approaches employed in the studies were assessed by principal component analysis.

The outcome ‘yearly citations received by a published paper’ was considered. A multivariable model was developed for the above outcome, and parameters found with *p* < 0.20 in the preceding univariable analyses were offered to this model (*n* = 8). Progressively, variables were removed from the model by using backward elimination. The likelihood ratio test was performed to assess the *p*-value of each parameter; among those found with *p* > 0.2, the one with the largest *p* was removed from the model. The procedure was repeated until no variable with *p* > 0.2 could be removed from the model. The variables included in the final multivariable models constructed are detailed in Table S1.

Statistical significance was defined at *p* < 0.05.

## 3. Results

### 3.1. Year of Publication of Papers

**Figure 1:**
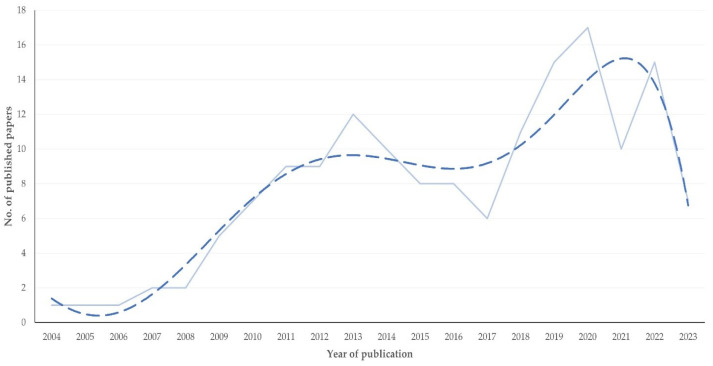
Number of papers on mastitis and proteomics published annually up to 2023 (dashed line indicates trendline).

The first relevant paper was published in 2004. Overall, there was a significant progressive increase in the number of articles published annually up to 2023 (slope ± standard error of the slope: 0.65 ± 0.12; *p* < 0.0001) (Figure 1). There was also an increase in the proportion of articles published annually compared to all articles on mastitis (*p* < 0.0001) and to all articles on proteomics (*p* = 0.044) (Table S2, Figure S1).

### 3.2. Origin of Papers

The 156 published papers originated from a total of 33 countries (Table S2); 17 (10.8%) papers originated from two different countries. The median number of published papers per country was 2 (interquartile range: 5). Of the 33 countries, 15 (45.5%) were European Union members.

**Figure 2:**
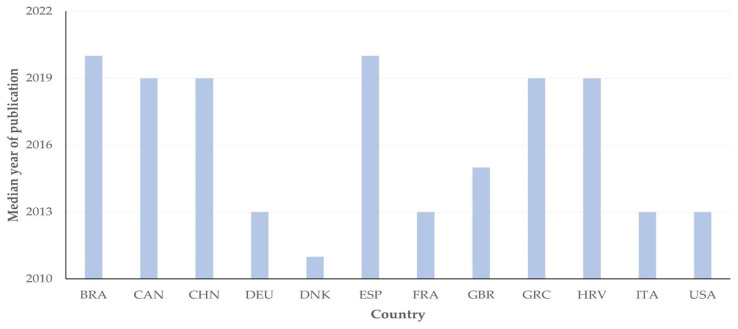
Median year of publication of papers on mastitis and proteomics from 12 countries with most published papers (≥5) on study topic (abbreviations of country names according to International Naming Convention ISO 3166 [11]).

There were 12 countries, from which at least five published papers originated. Among these, there was a significant difference in the median year of publication of papers; Denmark had the oldest median year of publication, whilst Brazil and Spain had the most recent median year of publication of relevant papers: 2011 (2) vs. 2020 (3) (*p* < 0.0001) (Figure 2).

**Figure 3:**
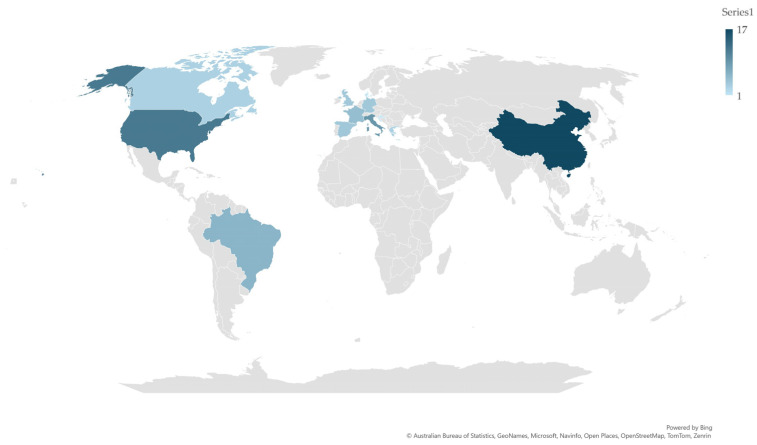
Number of scientific establishments, from which originated papers on mastitis and proteomics, among 12 countries with most published papers (≥5) on study topic (blue color palette in accord with number of scientific establishments within country).

Within these 12 countries, papers originated from a total of 69 scientific establishments (Table S3). Most of these (*n* = 49, 71.0%) were universities, with fewer ones (*n* = 20, 29.0%) being research institutes, national agencies or commercial entities (*p* < 0.0002). Among these, the median number of published papers per scientific establishment was 1 (3). There was some tendency for significance in the median number of papers published between universities and other types of scientific establishments: 1 (2) vs. 2 (5) (*p* = 0.08). The number of scientific establishments, from which relevant papers originated, varied from 1 (Croatia, Denmark) to 17 (China) per country (Figure 3). There was a significant correlation between the number of scientific establishments in a country and the number of papers that originated from that country (*r_sp_* = 0.88, *p* = 0.0002).

### 3.3. Content of Papers

Of the 156 published papers, most (*n* = 135, 86.6%) were original articles, and the remaining (*n* = 21, 13.4%) were reviews.

#### 3.3.1. Mammalian Species

**Figure 4:**
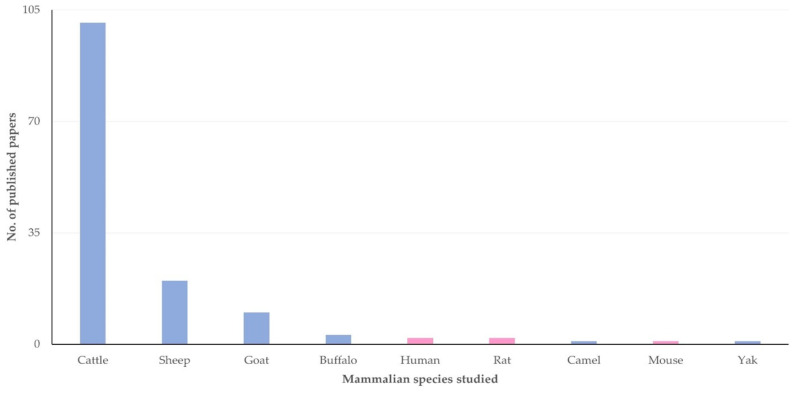
Number of original articles on mastitis and proteomics, in accord with mammalian species in which work was performed in respective studies.

Among the 135 original articles, most (*n* = 101, 74.8%) referred to work involving cattle; work involving another eight mammalian species was also described in the articles (Figure 4, Table S4).

**Figure 5:**
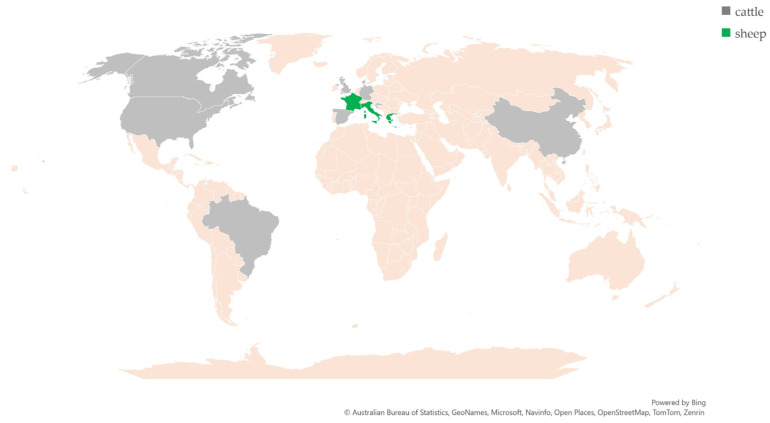
Mammalian species on which focused work described in majority of original articles on mastitis and proteomics.

There were differences among the above 12 countries in the number of papers published with regard to work in various mammalian species. Most articles that originated from France, Greece and Italy described work involving sheep, whilst articles from the other countries focused on work involving cattle (*p* = 0.0001) (Figure 5, Table S5, Figure S2).

#### 3.3.2. Mastitis Aspect and Type of Work

**Figure 6:**
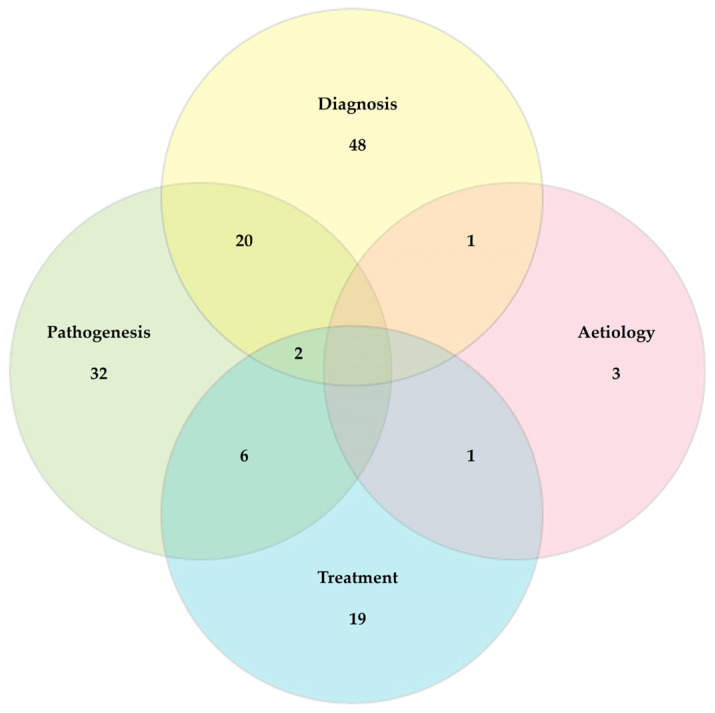
Venn diagram of mastitis aspect in respective studies among original articles on mastitis and proteomics (note: two papers on aetiology and pathogenesis concurrently and one paper on diagnosis and treatment concurrently could not be visualized).

Most original articles described work related to the diagnosis (*n* = 72; 53.3%) or pathogenesis (*n* = 62; 45.9%) of mastitis. Fewer articles described work on the treatment (*n* = 29; 21.5%) or aetiology (*n* = 7; 5.2%) of the infection (Figure 6).

Most original articles included field work (*n* = 75; 55.6%); fewer articles included experimental (i.e., challenge-associated) (*n* = 31; 23.0%) or laboratory (*n* = 30; 22.2%) work. There was a difference in the mammalian species involved in the type of such studies: the involvement of mice, rats and sheep was associated mainly with experimental work, whilst the involvement of buffaloes, cattle, camels, goats, humans and yaks was associated mainly with field work (*p* = 0.028) (Figure S3, Table S6); it is noted that one article presented natural infection in humans followed by experimental work in mice.

**Figure 7:**
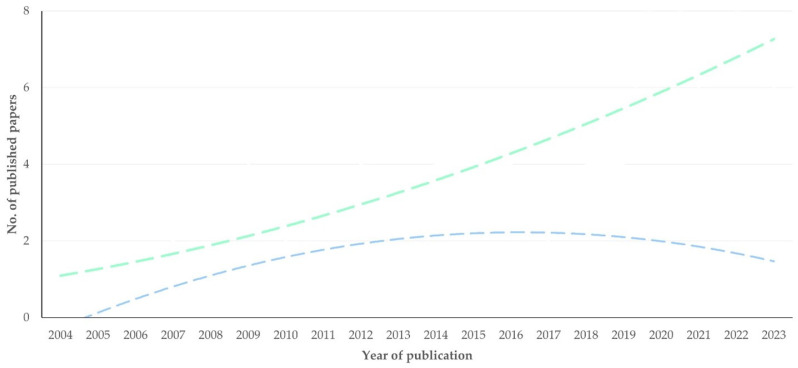
Trendlines for number of original articles on mastitis and proteomics describing experimental (blue) or field (green) work in respective studies, in accord with year of publication.

There was no significant difference in the median year of publication between articles presenting experimental (2015 (7.5)), field (2018 (9)) or laboratory-based (2017.5 (6)) work (*p* = 0.36) (Figure S4). However, there was a clear difference in the slope of the year of publication of original articles that described experimental (0.09 ± 0.05) or field (0.32 ± 0.06) work (*p* = 0.004) (Figure 7).

**Figure 8:**
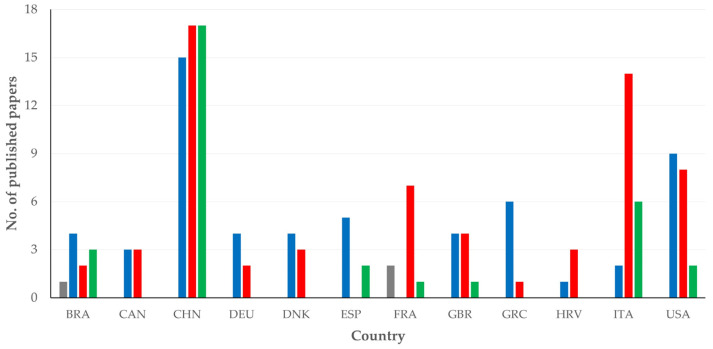
Number of original articles on mastitis and proteomics, in accord with country of origin and mastitis aspect in respective studies (abbreviations of country names according to International Naming Convention ISO 3166 [11]; gray bars: papers on aetiology, blue bars: papers on diagnosis, red bars: papers on pathogenesis, green bars: papers on treatment of mastitis).

There was also a significant difference between articles that originated from different countries regarding the mastitis aspect studied therein (*p* = 0.0003) (Figure 8, Table S7). For the diagnosis, pathogenesis and treatment of mastitis, most articles originated from China; specifically for treatment, articles from China accounted for 53.1% of all relevant papers.

#### 3.3.3. Material Assessed

The tissue assessed most frequently in the studies was the milk (*n* = 59, 43.7%); another three tissues, blood (*n* = 17), mammary tissue (*n* = 24) and saliva (*n* = 1), as well as milk fat globule (*n* = 4), were also assessed. Pathogens were assessed in studies described in 39 articles (28.9%) (Figure S5).

**Figure 9:**
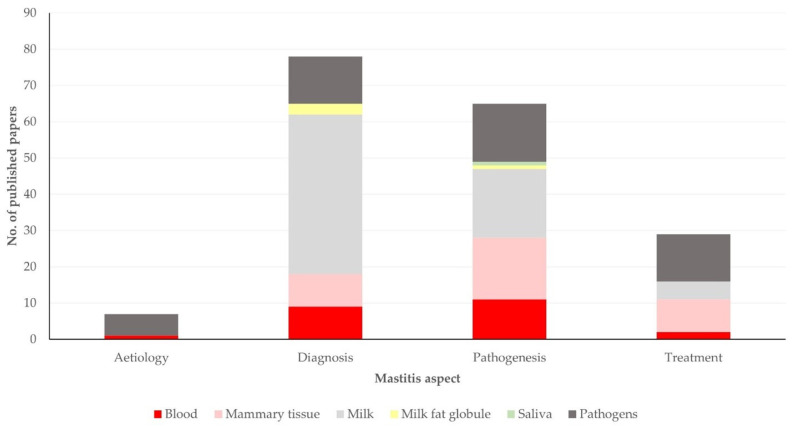
Number of original articles on mastitis and proteomics, in accord with material assessed and mastitis aspect studied in respective studies.

**Figure 10:**
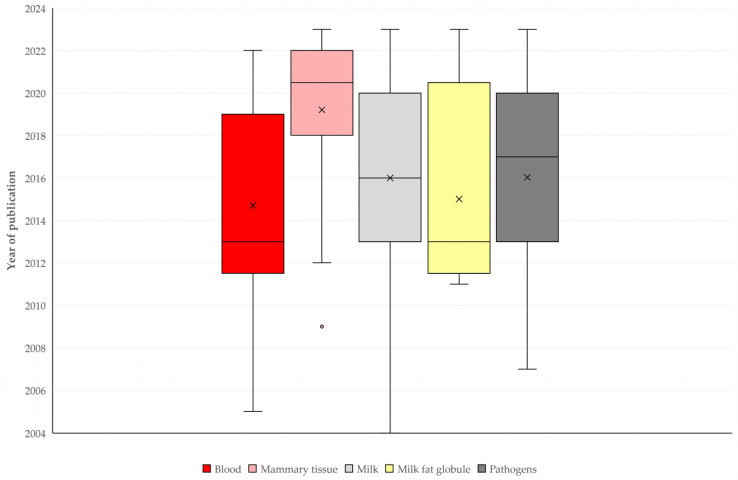
Box and whisker plot for year of publication of original articles on mastitis and proteomics, in accord with material assessed in respective studies.

There was a significant difference in the material assessed, in accord with the mastitis aspect studied: milk was assessed more frequently in studies on diagnosis (61.1% of relevant studies) or pathogenesis (30.6% of relevant studies) of the infection, but mammary tissue was assessed more frequently in studies on treatment (31.0%), whilst pathogens were assessed mainly in studies on the aetiology (85.7%) of mastitis (*p* = 0.0003) (Figure 9, Table S8). Further, a significant difference was seen in the median year of publication between articles, in accord with the material assessed (*p* = 0.014) (Figure 10).

In total, 47 pathogens (associated with mammary infection) were included in the studies described in the original article (Table S9). Most of these were Gram-positive bacteria (*n* = 34, 72.3%). Gram-negative bacteria (*n* = 8, 17.0%), algae (*n* = 2, 4.3%), fungi (*n* = 2, 4.3%) and protozoa (*n* = 1, 2.1%) were also included. The three bacteria most frequently included in the studies were *Staphylococcus aureus* (*n* = 55 articles), *Escherichia coli* (*n* = 31) and *Streptococcus uberis* (*n* = 19). No significant differences were found in the number of original articles that described studies in each of these three bacteria, with regard to the mammalian species (*p* = 0.77), mastitis aspect (*p* = 0.28), type of work (*p* = 0.69) and material assessed (*p* = 0.36) (Table S10).

#### 3.3.4. Proteomics Methodological Approaches

The proteomics methodological approaches employed in the various studies described in the original articles were clustered as detailed in Table 1. The proteomics technology employed more often in the respective studies was liquid chromatography-tandem mass spectrometry (LC-MS/MS), either on its own (*n* = 56 papers) or in combination with other technologies (*n* = 40 papers). A significant difference was seen in the median year of publication between articles, in accord with the proteomics methodological approach employed; the median year of publication of articles involving bioinformatics or LC-MS/MS with bioinformatics was the most recent, 2022 (interquartile ranges: 0 and 2, respectively), whilst the median year of publication of articles with two-dimensional difference gel electrophoresis (2D-DIGE), matrix-assisted laser desorption/ionization coupled to time-of-flight mass spectrometry (MALDI-TOF MS) and polyacrylamide gel electrophoresis followed by liquid chromatography-tandem mass spectrometry (GeLC-MS/MS) was the oldest: 2011 (3) (*p* < 0.0001).

**Figure 11:**
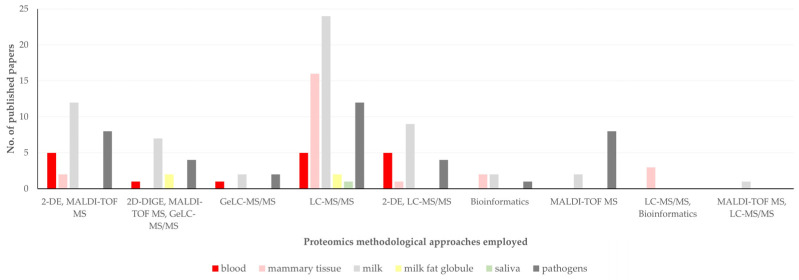
Original articles on mastitis and proteomics describing various proteomics methodological approaches employed, in accord with material assessed in respective studies.

There was also a significant difference in the proteomics methodological approach employed, in accord with the material assessed in each study (*p* = 0.020) (Figure 11, Table S11).

**Figure 12:**
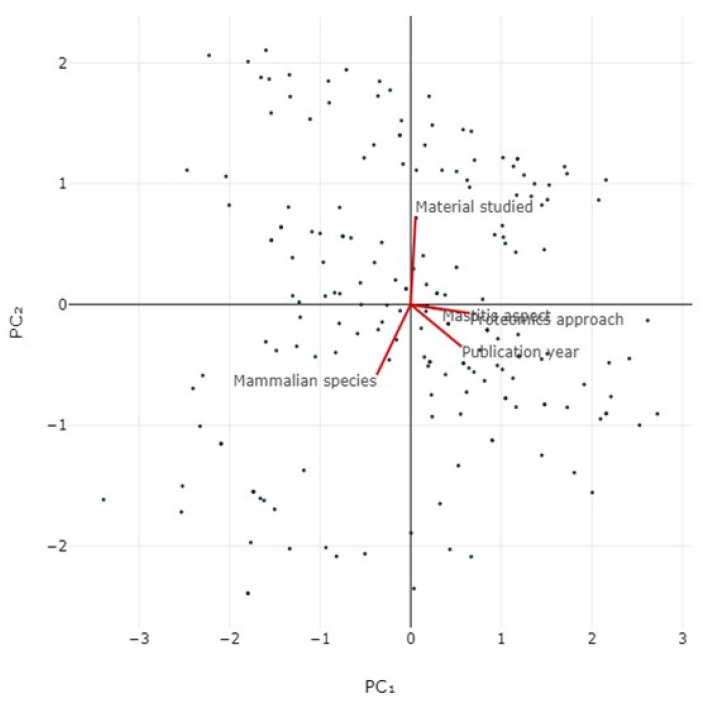
Bi-plot of results of principal component analysis for proteomics methodological approach, mammalian species, mastitis aspect, material assessed and year of publication in original articles on mastitis and proteomics.

Pairwise correlation analysis indicated a significant correlation between the proteomics methodological approach, mammalian species, mastitis aspect and year of publication (*r_sp_* > 0.14, *p* < 0.046) (Table S12). Principal component analysis for the proteomics methodological approach, mammalian species, mastitis aspect, material assessed and year of publication revealed that the two principal components accounted for 55.5% of the variation (Figure 12, Table S13, Figures S6 and S7).

A total of 132 combinations of proteomics methodological approaches, mammalian species, the mastitis aspect and the year of publication were found in the original articles (Table S14). The most frequent combination referred to the use of 2-DE and MALDI-TOF MS for the diagnosis of mastitis in sheep and was found in six papers published in 2019.

#### 3.3.5. Additional -Omics Technologies Described in Papers

In 25 original articles (18.5%), the study involved the use of additional -omics technologies. These referred to genomics (*n* = 15, 60.0%), transcriptomics (*n* = 7, 28.0%), peptidomics (*n* = 4, 16.0%), metabolomics (*n* = 2, 8.0%) and metagenomics (*n* = 1, 4.0%). Additional -omics techniques were employed more frequently in original articles, in which the material assessed was pathogens (*n* = 13, 52.0%) (*p* = 0.037) (Table S15).

### 3.4. Journals in Which Papers Were Published

The 156 papers were published in 78 different journals in total. The median number of papers published per journal was 1 (1) (Table S16). The journals (*n* = 8) in which at least five articles were published, were *Journal of Proteomics* (*n* = 16 papers), *Journal of Dairy Science* (*n* = 12), *Journal of Proteome Research* (*n* = 8), *International Journal of Molecular Sciences* and *Veterinary Microbiology* (*n* = 6 each) and *Animals*, *Data in Brief* and *Veterinary Research* (*n* = 5 each). In total, 63 papers (40.3%) were published in these eight journals.

**Figure 13:**
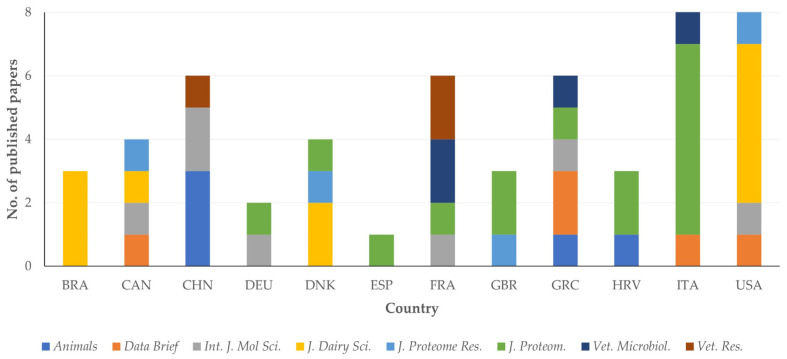
Association between journals and countries of origin of published papers on mastitis and proteomics (abbreviations of country names according to International Naming Convention ISO 3166 [11]; abbreviations of journals from left to right: *Animals*, *Data in Brief*, *International Journal of Molecular Sciences*, *Journal of Dairy Science*, *Journal of Proteome Research*, *Journal of Proteomics*, *Veterinary Microbiology*, *Veterinary Research*.

Cumulatively, 58 of these 63 papers (92.1%) originated from the 12 countries with most papers published. There were, however, differences among these countries in the journals, in which papers were published (*p* = 0.037) (Figure 13, Table S17).

Both original and review articles have been published in the following six journals: *Animals*, *International Journal of Molecular Sciences*, *Journal of Dairy Science*, *Journal of Proteomics*, *Pathogens* and *Veterinary Microbiology*.

The Web of Science sub-categories in which journals with published papers were classified, are in Table S18. The three sub-categories of journals, in which most papers were published, were Veterinary Sciences (*n* = 33), Biochemical Research Methods (*n* = 32) and Agriculture, Dairy and Animal Science (*n* = 25).

**Figure 14:**
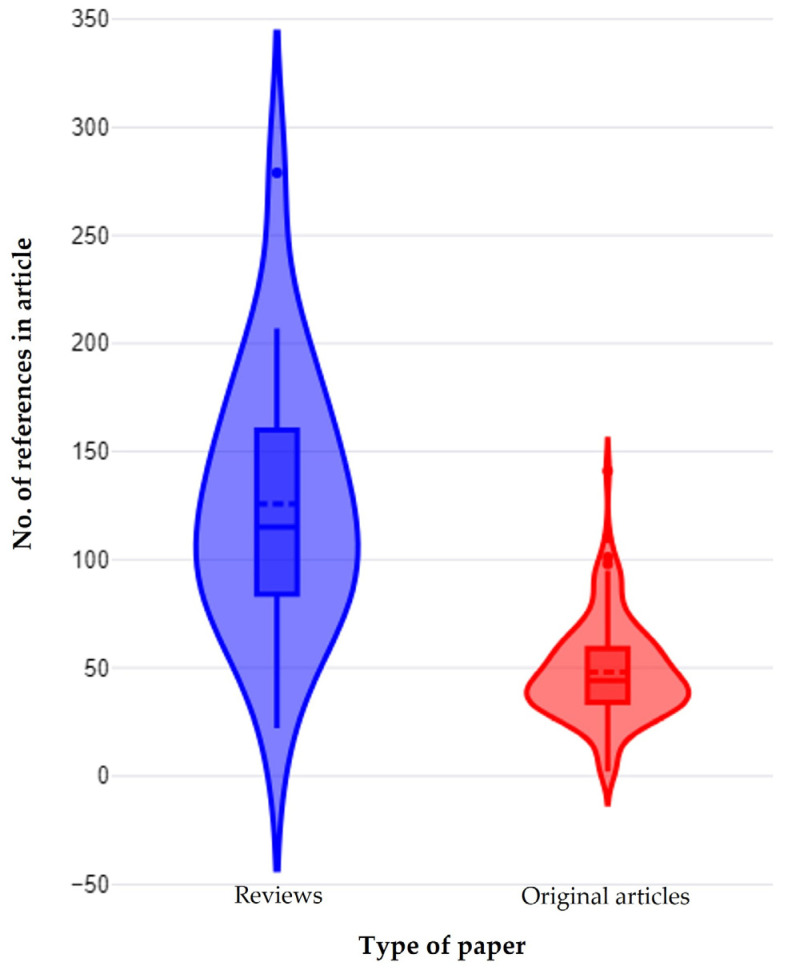
Violin plot for number of cited references in reviews and original articles on mastitis and proteomics.

The median number of cited references in the papers was 48 (interquartile range: 34). Reviews included a significantly higher number of cited references than original articles: 115 (76) vs. 44 (25) (*p* < 0.0001) (Figure 14). There was no correlation between the year of publication of the paper and the number of cited references therein (*r_sp_* = 0.042, *p* = 0.61).

### 3.5. Authors of Papers

Cumulatively, in the 156 papers, there were 1143 co-authors, i.e., on average 7.3 ± 0.3 co-authors per paper (median: 7 (4), min.–max.: 1–19). There were in total 742 individual authors of the papers. Among them, 15 authors had published at least seven papers (max.: 10).

Further, there were 218 individual authors who were first or last authors in the papers. Among these, 11 authors were first or last in at least four papers each (max.: 9).

The 15 authors with at least seven papers, were first or last authors in 45.6% ± 9.8% of their papers (min.: 0.0%, max.: 100.0%).

The 11 authors with at least four papers as first or last, were affiliated with scientific establishments in the 12 countries from which originated most published papers. However, it seemed there was a limited collaboration between these 11 authors. Among them, only three pairs of authors, affiliated with the same establishment in each of three different countries, were identified with joint papers (Figure S8). It is also noted that among these 11 authors, only one was among the 50 authors with the most published papers on mastitis.

There were differences between these 12 countries in the median number of authors per published paper. Papers from the United States of America had the smaller median number of authors, 4 (4.3) per paper, whilst papers from Croatia had the higher: 12 (4) (*p* = 0.0009 between countries) (Table S19).

**Figure 15:**
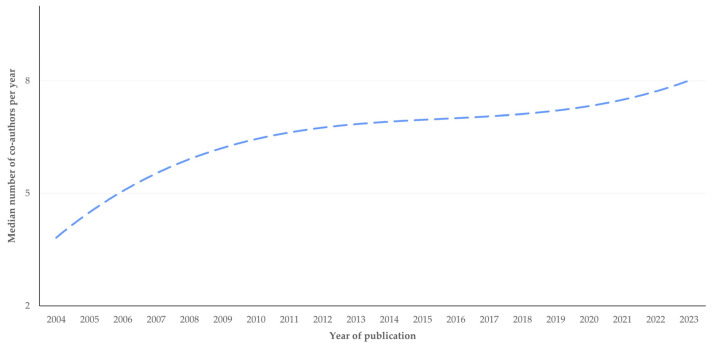
Change in median number of co-authors per year in papers on mastitis and proteomics through years (dashed line is trendline).

The average number of co-authors per published paper increased slightly throughout the years (slope: 0.167 ± 0.08) (*p* = 0.06) (Figure 15). In four papers (2.6%), there was only one author. Finally, the median number of authors in original papers was higher than in reviews: 8 (10) vs. 3 (5), respectively (*p* < 0.0001).

### 3.6. Accessibility of Papers

**Figure 16:**
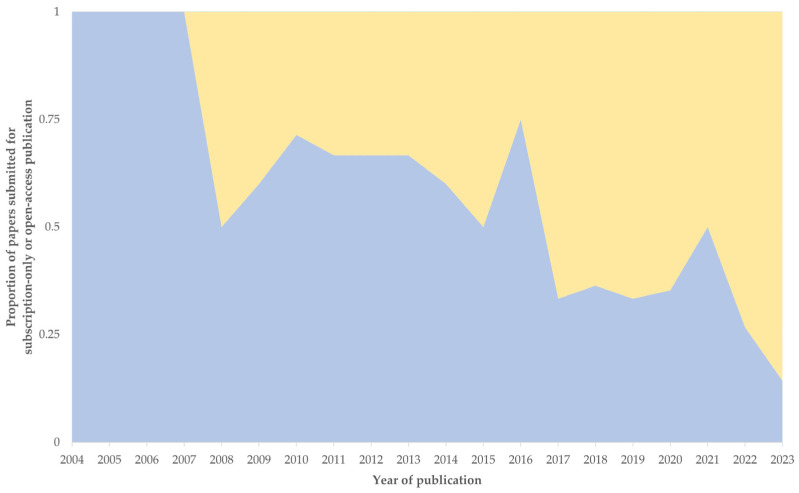
Yearly proportion of published papers on mastitis and proteomics, in accord with type of accessibility selection, i.e., submitted for subscription-only (blue) or for open-access (yellow) publication.

Among the 156 published papers, almost equal numbers were submitted for open-access publication or subscription-only access: 79 (50.6%) vs. 77 (49.4%). However, the median year of publication of the former papers was significantly more recent than that of the latter ones: 2019 (6) vs. 2014 (8) (*p* < 0.0001); indeed, the proportion of the latter papers progressively decreased (Figure 16).

The proportion of papers submitted for open-access publication was highest among papers from Croatia (80.0%) and lowest among papers from the United States of America (30.0%) (*p* = 0.47 between countries).

### 3.7. Impact of Papers

The median number of citations received by the 156 papers was 12 (interquartile range: 25) (min.–max.: 0–339), and the median yearly number of citations was 2.0 (2.6) (min.–max.: 0.0–29.5). The *h*-index of the papers was 33, and the *m*-index was 2.

**Figure 17:**
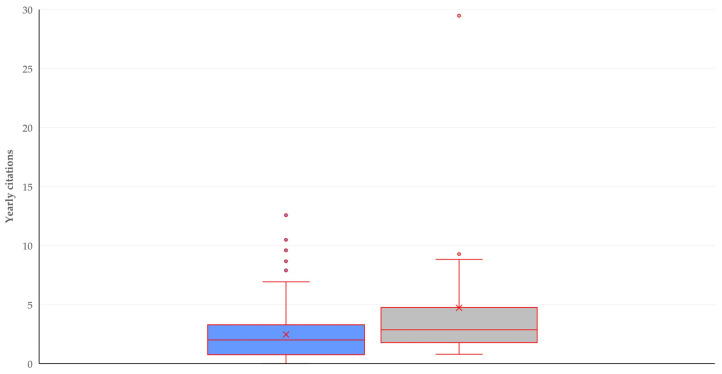
Box and whisker plot for yearly citations received by original articles (blue) or reviews (gray) on mastitis and proteomics.

There was no difference in the number of yearly citations received by papers published under open- or subscription-only access, 2.0 (2.3) vs. 2.4 (3.2) (*p* = 0.21), whilst there was a tendency for correlation between the yearly number of citations and the number of authors (*r_sp_* = 0.153, *p* = 0.06). Overall, there was a clear correlation between the yearly number of citations and the number of cited references (*r_sp_* = 0.386, *p* < 0.0001); this was found for original articles (*r_sp_* = 0.352, *p* < 0.0001) but not for reviews (*r_sp_* = 0.197, *p* = 0.39). This latter type of paper received a higher number of yearly citations than original articles: 2.9 (2.4) vs. 2.0 (2.4) (*p* = 0.020) (Figure 17).

With regard to the country of origin of the papers, those that originated from two different countries (i.e., with an international collaboration) had received more yearly citations than those from a single country: 2.8 (5.1) vs. 2.0 (2.3) (*p* = 0.044). There were also differences between the published papers in the number of yearly citations according to their country of origin (*p* = 0.06) (Table S20). Finally, with regard to the content of the paper, there was no significant association for the yearly number of citations with any of the variables evaluated (*p* ≥ 0.08 for all comparisons) (Table S21).

**Figure 18:**
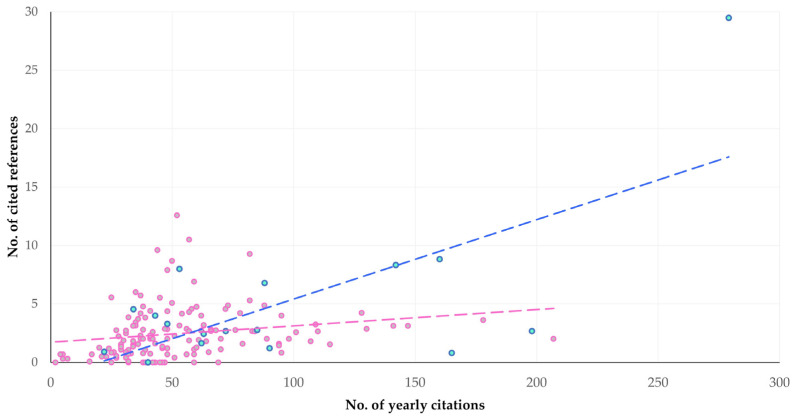
Trendlines for cross-plot of number of cited references and respective yearly citations for published papers on mastitis and proteomics, with (green-blue) or without (gray-pink) international collaboration.

During the multivariable analysis, a significant association of yearly citations was found with the number of cited references (*p* < 0.0001), whilst a tendency was also seen for papers with international collaboration (*p* = 0.06) (Table 2, Figure 18).

## 4. Discussion

### 4.1. Year of Publication

Proteomics technologies have evolved during the last 20 years. Proteomics capitalized on the completion of major genomics projects around the beginning of the century and the development of mass spectrometry and computational tools. The technologies have been applied initially for the study of various human diseases; subsequently, their use has been extended to evaluating samples of veterinary importance.

In relation to the study of mastitis, initially, proteomics technologies have been implemented primarily in the United States of America [12,13,14,15] and also in the United Kingdom [16]. These were followed by groups in Italy [17] and New Zealand [18]. Those studies focused on using proteomics technologies to deepen research on mastitis pathogenesis in cattle.

Progressively, as proteomics trends evolved globally, the potential use of the technologies for the diagnosis of the infection has developed, through work for the discovery of potential biomarkers [19,20,21]. Moreover, work has been extended to include dairy small ruminants, sheep [22] and goats [23].

Thereafter, the use of the technologies has steadily increased. It is notable that a Cost Action on ‘Farm animal proteomics’ was implemented in the European Union from 2010 to 2014 (reference FA 1002). This furthered involvement of researchers in the broad field of work and increased the number of relevant publications. As part of this action, a special issue was also published in the *Journal of Proteomics* [24].

All these are aligned with the increase in the number of relevant published papers that was seen during the 2010s. Nevertheless, the results indicated that the number of published papers in recent years appeared to have stabilized, rather than increasing further, especially in comparison to the increase in papers published on mastitis alone or proteomics alone. This can reflect possibly a diversion of research resources to other priorities subsequently to the COVID-19 pandemic in the early 2020s [25], but it may also be the result of reaching the potential limits of use of the technology in mastitis research, as a consequence of data saturation in the topic [26].

### 4.2. Countries of Origin

It is evident, based on the research output, that relevant work has originated from three major parts of the world: China, the European Union and the United States of America.

During recent years, research and development in China have surged, and the allocation of state-derived funding has increased [27,28]. This can be seen in the increase in research output from Chinese scientific establishments [29], which is aligned with the evidence from the present assessment. The focus on the treatment of mastitis observed among articles that originated from China can reflect the increasing pharmaceutical work carried out in this country, the investments made in that sector and the new legal framework for drug licensing established in this country [30].

With regard to the European Union countries, the Cost Action mentioned above has contributed to increased involvement in applying proteomics in mastitis work. That action involved a large number of memberstates, which has been reflected in the publication of relevant papers from 15 countries of the Union. It is interesting that relevant studies from France, Greece and Italy have prioritized research on sheep rather than cattle as did other countries of the world; this reflects the importance of oviculture as a sector of animal production in these countries.

The United States of America is the origin of most published papers on mastitis (21.6%) and proteomics (32.7%) and hence also has a large number of research papers on the combined topics. In this country, the technological background available for research [31] coupled with the significance of the dairy sector (accounting for 3.5% of the total GDP [32]) have contributed to applying the technologies for improving dairy production.

All the above are also reflected in the higher number of scientific establishments from which relevant papers originated, in the above three parts of the world.

### 4.3. Mastitis Content

Most original articles presented work performed on cattle. Milk is a valuable agricultural commodity globally; it is among the top five agricultural commodities worldwide. Cow milk accounts for 80% to 85% of global milk production [33], and hence most relevant studies were performed on cattle.

Protein interactions and the discovery of molecular pathways through the course of a disease are key principles of proteomics [34]. In this regard, the discovery of potential biomarkers for the early diagnosis of diseases is part of the main objectives of the technologies [34]. Moreover, some articles studied more than one mastitis aspect. In contrast, the small number of articles on the aetiology of the infection reflects that proteomics is not widely used for pathogen identification; articles on proteomics and mastitis aetiology referred to the use of proteomics technologies (MALDI biotyping) for the identification of pathogens [35,36,37].

The progressive decrease in experimental studies is likely the consequence of concerns over animal welfare and more strict legislation on animal use in research, as well as possibly the increased costs associated with challenge studies (e.g., expenses for infrastructure, for animals and animal maintenance, etc.) [38]. Experimental studies were performed more often in sheep than other animal species, which may be the result of the specific research interests of the various groups working on the topic; nevertheless, it is noted also that sheep can be valuable as models for some aspects of cattle mastitis research [39,40], given the significant differences in maintenance costs between the two animal species, due to the easier management and handling of sheep compared to cattle.

Initially, the samples used in the studies were blood and milk, i.e., tissues easily collected by means of no invasive techniques, processed by technologies available in the 2000s and 2010s and useful for biomarker discovery. The collection of mammary tissue is tedious, and its processing by proteomics technologies poses difficulties [41,42]. The interest in furthering pathogenesis studies required the analysis of mammary tissue samples, which kept pace with the evolution of relevant technologies and the development of LC-MS/MS; this is in line with the observation of more recent relevant publications and the increased use of this technology for assessing mammary tissue.

### 4.4. Proteomics Methodologies

Various methodologies for studying proteomes have emerged and continue to evolve rapidly. Mass spectrometry is the most frequently and widely employed technology in proteomics work for protein identification. Various technical advancements (e.g., improved capillaries, higher flow rate, more robust columns, better reproducibility) that have occurred throughout the years, have allowed improved processing of samples and materials under assessment [43]. Nevertheless, the preparation and deposition of samples into the mass spectrometer target for evaluation, as it is applied in MALDI MS, and the interference from matrix ions are considered limitations [44].

Material assessed in earlier studies included blood and milk (as shown by the year of publication of relevant studies). However, in more recently published papers, the assessment of mammary tissue has increased. Possibly, the technical advancements in using LC-MS/MS in recent years have contributed to the increasing assessment of mammary tissue, which could provide more in-depth information than those obtained by assessing other material. The use of mammary tissue would likely contribute better to the elucidation of the pathogenesis of the infection.

The findings also indicate that the combination of two-dimensional gel electrophoresis and MALDI-TOF/MS is still employed in relevant studies. The approach can be used for the proteoform separation and identification approach [45]. Proteoforms (or ‘protein species’) refer to the different forms of proteins produced from the genome with a variety of sequence variations, splice isoforms and many post-translational modifications and are important in various biological systems [40,45]. Moreover, with this approach, one can achieve a semi-quantification of proteins on the gels [46]. In general, gel-based methods and techniques employed in earlier studies have some shortcomings; hence, in order to identify the dynamic range and quantitative accuracy of the results, gel-free and more sensitive high-throughput quantitative proteomics methods have been applied in mastitis research [47].

The field of bioinformatics has emerged and developed revolving around proteomics inquiries. Bioinformatics encompasses the entirety of biological information, which refers to the acquisition, processing, storage, dissemination, analysis, and interpretation of large sets of biological data. By combining biological and computational tools, it can become possible to elucidate and understand the biological importance within such large sets [48]. The use of bioinformatics in proteomics studies aligns with the variety of strategies and methodologies employed in proteomics research, for example, the processing of data derived from mass spectrometry, given that a mass spectrometer can generate millions of spectra within a relatively short period [49]. For the analysis of such large datasets, the availability of efficient and user-friendly computational tools is paramount [49,50,51]. This development of bioinformatics has resulted in its increased use in proteomics work, as shown in the recent published papers.

The use of additional -omics technologies (most frequently genomics) in papers that studied pathogens associated with mastitis possibly indicates a multi-omics approach, rather than a proteomics-focused study. For example, some of these papers studied pathogen secretome; this includes a number of bacteria-released factors with a variety of roles (e.g., virulence factors) [52], thus playing a role in the pathogenesis of the infection.

### 4.5. Bibliometric Details

Many papers were published in journals with a specific approach to proteomics work (e.g., *Journal of Proteomics*, *Journal of Proteome Research*, *Proteomics*) or journals with a thematic approach to animal studies (e.g., *Journal of Dairy Science*, *Animals*, *Animal*) or journals focusing on animal infections (e.g., *Veterinary Microbiology*, *Veterinary Research*, *Frontiers in Microbiology*). In contrast, few papers were published in multidisciplinary journals (e.g., *PLoS ONE*, *Scientific Reports*, *PeerJ*), although the topic was clearly of multidisciplinary interest. This indicates the increased significance of thematic journals and the preference of research workers to publish in them; this indicates that researchers prefer to present their research output to a specialized audience with a focused interest in the same topic and the ability to comprehend it.

Interest in using proteomics in veterinary research and clinical work has been growing, but veterinarians are still not fully familiar with the possibilities of the technology. It seems that specific research groups active in mastitis research have teamed up with groups active in proteomics work and have set up lasting collaborations. This may explain the small increase through the years in the number of authors among the published papers, although there is a global trend for increasing the number of co-authors in published papers, across all scientific disciplines [53]. The opportunities for international collaboration across the European Union countries, possibly as the result of the Cost Action mentioned above, may also have accounted for the higher number of authors per published papers shown in papers of origin from European countries.

The increasing proportion of papers published under open access aligns with the current trends for the publicity of scientific results, especially of findings derived from studies carried out with public grants [54]. The increasing proportion of open-access publications seen is in line with the general evidence available internationally regarding the expansion of open-access publishing [55]. Open-access publishing promotes scientific communication and dissemination of scientific knowledge; it is the means by which research findings become available free of charge to the scientific community and the wider public [56].

The finding for more citations of the papers with longer lists of cited references aligns with previous findings, which relate to papers across the board of scientific disciplines [57,58,59,60,61]. One may suggest that a large number of references in a published paper is the result of such a paper dealing with a variety of issues and presenting a diversity of ideas, which makes it more easily citable, as it includes more information. Moreover, such papers may be more easily retrieved in online searches on document platforms [61]. International collaborations (which were also found to be associated with a higher number of citations) can allow additional visibility of published papers, as well as the use of more complex methodologies that lead to producing results of wider interest.

## 5. Conclusions

In conclusion, this is the first-ever scientometrics evaluation of proteomics studies. The results highlighted the characteristics of the papers assessed in the study. The use of proteomics in mastitis research has focused on the elucidation of pathogenesis and diagnosis of the infection. LC-MS/MS has been established as the most frequently used proteomics technology, although the use of bioinformatics has also emerged recently as a useful tool. Despite the multidisciplinary content of the papers, these have appeared mainly in topical scientific journals. The increased number of cited references in papers and international collaboration in the respective study were the variables associated with most citations to published papers.

## Figures and Tables

**Table 1:** Proteomics methodological approaches described in original articles on mastitis and proteomics.

Proteomics Methodological Approaches ^1^	No. of Articles	Median Year of Publication
LC-MS/MS	56	2019
2-DE, MALDI-TOF MS	24	2015
2-DE, LC-MS/MS	18	2015
2D-DIGE, MALDI-TOF MS, GeLC-MS/MS	13	2011
MALDI-TOF MS	10	2021
GeLC-MS/MS	5	2013
Bioinformatics	5	2022
LC-MS/MS, Bioinformatics	3	2022
MALDI-TOF MS, LC-MS/MS	1	2020

^1^ LC-MS/MS: liquid chromatography-tandem mass spectrometry, 2-DE: two-dimensional gel electrophoresis, MALDI-TOF MS: matrix-assisted laser desorption/ionization coupled to time-of-flight mass spectrometry, 2D-DIGE: two-dimensional difference gel electrophoresis, GeLC-MS/MS: polyacrylamide gel electrophoresis followed by liquid chromatography-tandem mass spectrometry.

**Table 2:** Results of multivariable analysis for variables with significant association with yearly citations of published papers on mastitis and proteomics.

Variables	Odds Risk (±se) ^1^	*p*
Cited references in papers		<0.0001
Per unit increase	1.040 ± 1.008	-
International collaboration		0.06
No (2.0 (2.3) ^2^)	reference	-
Yes (2.8 (5.1))	4.096 ± 2.115	-

^1^ se: standard error; ^2^ median (interquartile range) number of yearly citations.

## Data Availability

All data are available on the Web of Science platform and in the Supplementary Materials (www.webofknowledge.com).

## Supplementary Materials

The following supporting information can be downloaded at https://www.mdpi.com/article/10.3390/pathogens13040324/s1, Table S1: Details of multivariable model employed for evaluation of predictors for yearly citations of papers on mastitis and proteomics; Table S2: Number of papers on mastitis and proteomics published annually from 1971 to 2023; Figure S1: Proportion of published papers on mastitis and proteomics shown as proportion of all papers on mastitis or proteomics; Table S3: Number of published papers on mastitis and proteomics, in accord with country of origin; Table S4: Scientific establishments in the 12 countries with most (≥5) published papers on mastitis and proteomics and respective number of papers from these; Table S5: Number of published papers on mastitis and proteomics, in accord with mammalian species involved in respective studies; Table S6: Number of original articles on mastitis and proteomics, in accord with country of origin and animal species involved in respective studies; Figure S2: Number of original articles on mastitis and proteomics in accord with country of origin and animal species involved in respective studies; Figure S3: Number of original articles on mastitis and proteomics in accord with mammalian species involved in the study and type of work performed in respective studies; Table S7: Number of original articles on mastitis and proteomics by type of work referred to therein (experimental work or field work) and in accord with mammalian species involved in respective studies; Figure S4: Box and whisker plot for year of publication of original articles on mastitis and proteomics by type of work in respective studies: experimental work, field work or laboratory-based work; Figure S5: Number of original articles on mastitis and proteomics, in accord with material assessed in respective studies; Table S8: Number of original articles on mastitis and proteomics, in accord with country of origin and mastitis aspect in respective studies; Table S9: Number of original articles on mastitis and proteomics, in accord with material assessed and mastitis aspect in respective studies; Table S10: Pathogens included in studies on mastitis and proteomics and number of original articles describing their evaluation in respective studies; Table S11: Number of original articles on mastitis associated with *Escherichia coli* or *Staphylococcus aureus* or *Streptococcus uberis* and proteomics, in accord with study details; Table S12: Number of original articles with various proteomics methodological approaches, in accord with material assessed in respective studies; Table S13: Results of pairwise correlation analysis (*r_sp_*) for proteomics methodological approach, mammalian species, mastitis aspect, material assessed and year of publication described in original articles on mastitis and proteomics; Table S14: Eigenvalues for principal component analysis for proteomics approach, mammalian species, mastitis aspect, material assessed and year of publication described in original articles on mastitis and proteomics; Figure S6: Screeplot of results of principal components analysis for proteomics methodological approach, mammalian species, mastitis aspect, material assessed and year of publication in original articles on mastitis and proteomics; Figure S7: Ternary plot of results of principal components analysis for proteomics approach, mammalian species, mastitis aspect, material assessed and year of publication described in papers on mastitis and proteomics; Table S15: Heat plot of combinations of proteomics methodological approach, mammalian species, mastitis aspect and year of publication described in original articles on mastitis and proteomics; Table S16: Original articles on mastitis and proteomics, describing involvement of additional -omics technologies, in accord with material assessed in respective studies; Table S17: Journals in which papers on mastitis and proteomics were published and respective number of papers; Table S18: Association between journals and countries of origin of published papers on mastitis and proteomics; Table S19: Sub-categories of journals in Web of Science, in which papers on mastitis and proteomics were published, and respective number of papers; Figure S8: Venn diagrams of three pairs of authors, affiliated with scientific establishments in three different countries, with number of published papers in which they were first or last authors (descriptors of authors not corresponding to their names); Table S20: Median (interquartile range) number of authors per published paper on mastitis and proteomics, in accord with countries of origin of the papers; Table S21: Median (interquartile range) number of yearly citations received by published papers on mastitis and proteomics, in accord with countries of origin of papers; Table S22: Statistical significance of evaluation for potential association of variables of paper content with yearly number of citations.
