# Supplementary material for: Scientometrics Evaluation of Published Scientific Papers on the Use of Proteomics Technologies in Mastitis Research in Ruminants
Source: Pathogens. 2024 Apr 15;13(4):324. doi: 10.3390/pathogens13040324 (PMC11053840; doi:10.3390/pathogens13040324)
Supplement: Supplementary file 1 [file pathogens-13-00324-s001.zip › pathogens-2928390-supplementary.pdf]

# Scientometrics Evaluation of Published Scientific Papers on the Use of Proteomics Technologies in Mastitis Research

**Maria V. Bourganou, Dimitris C. Chatzopoulos, Daphne T. Lianou, George Th. Tsangaris, George C. Fthenakis and Angeliki I. Katsafadou**

**Table S1.** Details of a multivariable model employed for evaluation of predictors for yearly citations of papers on mastitis and proteomics.

| Outcome | Variables offered to the multivariable model | Variables required in the final test |
|---|---|---|
| Yearly citations of papers on mastitis and proteomics | $n = 8$ | (a) type of paper (original article or review), (b) country of origin of paper, (c) international collaboration in the origin of the article, (d) no. of cited references in paper |

**Table S2.** Number of papers on mastitis and proteomics published annually from 1971 to 2023.

| Year of paper publication | No. of papers published annually on mastitis | No. of papers published annually on proteomics | No. of papers published annually on mastitis and proteomics |
|---|---|---|---|
| 1971-2003 | 190 (annual mean) | 150 (annual mean) | 0 |
| 2004 | 468 | 2824 | 1 |
| 2005 | 467 | 3356 | 1 |
| 2006 | 510 | 4140 | 1 |
| 2007 | 550 | 4513 | 2 |
| 2008 | 493 | 4873 | 2 |
| 2009 | 620 | 5425 | 5 |
| 2010 | 639 | 6111 | 7 |
| 2011 | 716 | 6445 | 9 |
| 2012 | 798 | 7237 | 9 |
| 2013 | 816 | 7163 | 12 |
| 2014 | 729 | 7269 | 10 |
| 2015 | 831 | 7711 | 8 |
| 2016 | 818 | 8069 | 8 |
| 2017 | 810 | 8177 | 6 |
| 2018 | 945 | 8380 | 11 |
| 2019 | 1084 | 9682 | 15 |
| 2020 | 1264 | 10886 | 17 |
| 2021 | 1384 | 12283 | 10 |
| 2022 | 1279 | 12362 | 15 |
| 2023 | 1206 | 12335 | 7 |
| slope ± s.e. 2004-2023 | 46.14 ± 3.67 [a,b] | 483.01 ± 22.90 [a,c] | 0.65 ± 0.12 [b,c] |
| slope ± s.e. 2017-2023 | 77.00 ± 24.29 [a,b] | 822.82 ± 110.61 [a,c] | 0.21 ± 0.87 [b,c] |

[a-c]: same letters indicate $p < 0.0001$ between respective slopes.

**Figure S1.** Proportion of published papers on mastitis and proteomics shown as proportion of all papers on mastitis (left graph) or on proteomics (right graph) (dashed lines indicate respective trendlines).

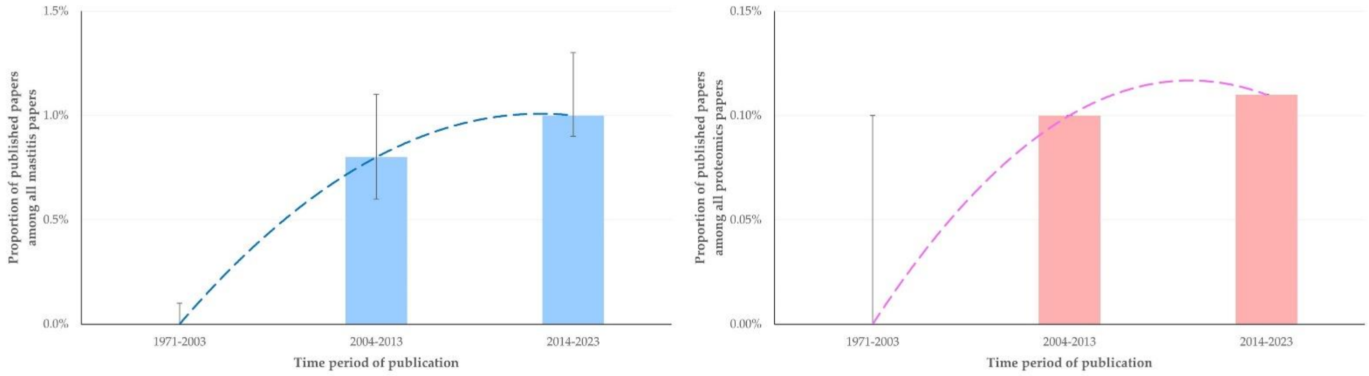

**Table S3.** Number of published papers on mastitis and proteomics, in accord with country of origin.

| Country | No. of papers published |
|---|---|
| China | 35 |
| United States of America | 20 |
| Italy | 19 |
| Brazil | 10 |
| Greece | 10 |
| France | 9 |
| United Kingdom | 9 |
| Spain | 7 |
| Canada | 6 |
| Germany | 6 |
| Croatia | 5 |
| Denmark | 5 |
| India | 4 |
| New Zealand | 3 |
| Portugal | 3 |
| Colombia | 2 |
| Egypt | 2 |
| Finland | 2 |
| Ireland | 2 |
| The Netherlands | 2 |
| Argentina | 1 |
| Australia | 1 |
| Czech Republic | 1 |
| Hungary | 1 |
| Japan | 1 |
| Korea | 1 |
| Norway | 1 |
| Pakistan | 1 |
| Poland | 1 |
| Russia | 1 |
| Saudi Arabia | 1 |
| Thailand | 1 |
| Turkey | 1 |

**Table S4.** Scientific establishments in the 12 countries with most (≥ 5) published papers on mastitis and proteomics and respective number of papers from these.

| Scientific establishment | Country [1] | No. of papers published |
|---|---|---|
| University of Thessaly | GRC | 10 |
| University of Milan | ITA | 9 |
| US Department of Agriculture | USA | 9 |
| Gansu Agricultural University | CHN | 8 |
| Academy of Athens | GRC | 7 |
| Porto Conte Research Institute | ITA | 7 |
| University of Glasgow | GBR | 7 |
| Agrocampus Ouest | FRA | 6 |
| Anhui Academy of Agricultural Sciences | CHN | 6 |
| Chinese Academy of Agricultural Sciences | CHN | 6 |
| Food and Drug Administration | USA | 6 |
| French National Research Institute for Agriculture, Food and Environment | FRA | 6 |
| Aarhus University | DNK | 5 |
| University of Zagreb | HRV | 5 |
| Beijing University of Agriculture | CHN | 4 |
| National Agency for Food, Environmental and Occupational Health & Safety | FRA | 4 |
| Northeast Agricultural University | CHN | 4 |
| University of Guelph | CAN | 4 |
| University of Santiago de Compostela | ESP | 4 |
| Federal University of Minas Gerais | BRA | 3 |
| Free University of Berlin | DEU | 3 |
| Sao Paulo State University | BRA | 3 |
| University of Sassari | ITA | 3 |
| Federal Rural University of Rio de Janeiro | BRA | 2 |
| Institute for Systems Biology | USA | 2 |
| Institute for the Animal Production System in the Mediterranean Environment | ITA | 2 |
| Mediterranean Center For Disease Control | ITA | 2 |
| Sichuan Agricultural University | CHN | 2 |
| University of California, Davis | USA | 2 |
| University of Clermont Auvergne | FRA | 2 |
| University of Padua | ITA | 2 |
| Yangzhou University | CHN | 2 |
| Zhejiang University | CHN | 2 |
| Agricultural University of Athens | GRC | 1 |
| Animal & Plant Health Agency | GBR | 1 |
| Boise State University | USA | 1 |
| Chinese Academy of Sciences | CHN | 1 |
| Cornell University | USA | 1 |
| Federal University of Santa Maria | BRA | 1 |
| Federal University of Vicosa | BRA | 1 |
| Fluminense Federal University | BRA | 1 |
| Guanxi University | CHN | 1 |
| Hipra Scientific Inc. | ESP | 1 |

| | | |
|---|---|---|
| Huazhong Agricultural University | CHN | 1 |
| JAM Council | FRA | 1 |
| Jiangsu Academy of Agricultural Sciences | CHN | 1 |
| Kingston University | GBR | 1 |
| Lanzhou University | CHN | 1 |
| Ludwig Maximilians Univesity of Munich | DEU | 1 |
| McGill University | CAN | 1 |
| Nanjing Agricultural University | CHN | 1 |
| Polytechnic of Milan | ITA | 1 |
| Precigen Inc. | USA | 1 |
| Rockefeller University | USA | 1 |
| Shandong Academy of Agricultural Sciences | CHN | 1 |
| Shihezi University | CHN | 1 |
| Texas Tech University | USA | 1 |
| University of Alberta | CAN | 1 |
| University of Bonn | DEU | 1 |
| University of Greifswald | DEU | 1 |
| University of Maryland | USA | 1 |
| University of Murcia | ESP | 1 |
| University of Naples Federico II | ITA | 1 |
| University of Nottingham | GBR | 1 |
| University of Perugia | ITA | 1 |
| University of Zaragoza | ESP | 1 |
| Virginia Tech | USA | 1 |
| Waksman Institute of Microbiology | USA | 1 |
| Zhejiang University of Science and Technology | CHN | 1 |

[1] abbreviations of country names according to International Naming Convention ISO 3166.

**Table S5.** Number of published papers on mastitis and proteomics, in accord with mammalian species involved in respective studies.

| Mammalian species | No. of published papers |
|---|---|
| Cattle | 101 |
| Sheep | 20 |
| Goat | 10 |
| Buffalo | 3 |
| Human | 2 |
| Rat | 2 |
| Camel | 1 |
| Mouse | 1 |
| Yak | 1 |

**Table S6.** Number of original articles on mastitis and proteomics, in accord with country of origin and animal species involved in respective studies.

| Country [1] | Animal species | | | | | |
|---|---|---|---|---|---|---|
| | Buffalo | Cattle | Goat | Rat | Sheep | Yak |
| Brazil | 2 | 6 | 1 | 0 | 0 | 0 |
| Canada | 0 | 5 | 0 | 0 | 0 | 0 |
| China | 0 | 29 | 1 | 2 | 1 | 1 |
| Croatia | 0 | 3 | 0 | 0 | 0 | 0 |
| Denmark | 0 | 4 | 0 | 0 | 0 | 0 |
| France | 0 | 3 | 0 | 0 | 5 | 0 |
| Germany | 0 | 5 | 0 | 0 | 0 | 0 |
| Greece | 0 | 1 | 0 | 0 | 7 | 0 |
| Italy | 1 | 5 | 4 | 0 | 6 | 0 |
| Spain | 0 | 7 | 0 | 0 | 0 | 0 |
| United Kingdom | 1 | 5 | 1 | 0 | 0 | 0 |
| United States of America | 0 | 15 | 2 | 0 | 0 | 0 |

[1] only the 12 countries with most (≥ 5) published papers are included.

**Figure S2.** Number of original articles on mastitis and proteomics in accord with country of origin [1] and animal species involved in respective studies.

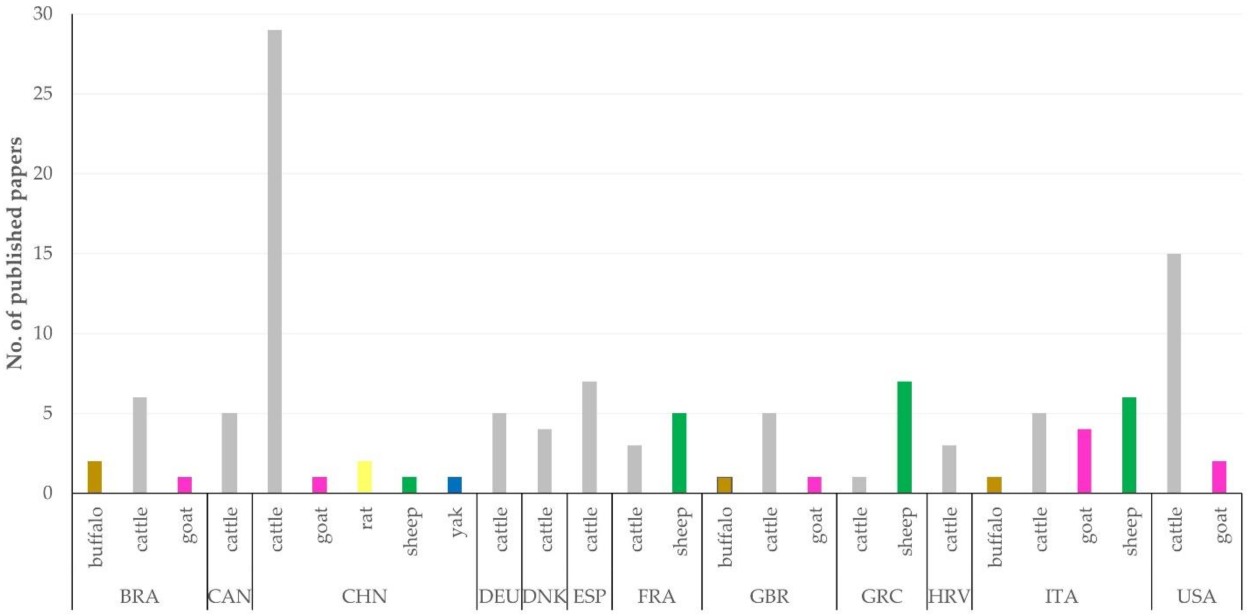


[1] only the 12 countries with most (≥ 5) published papers are included.

**Figure S3.** Number of original articles on mastitis and proteomics in accord with mammalian species involved in the study and type of work (experimental: blue - field: green) performed in respective studies.

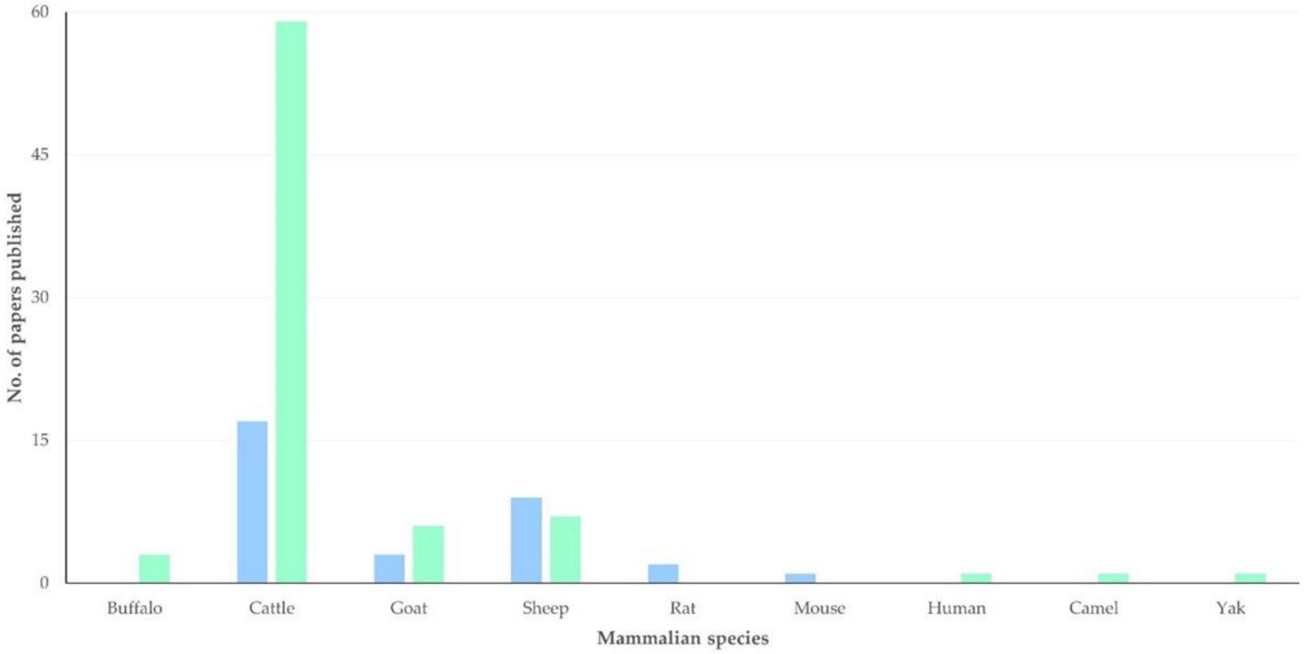

**Table S7.** Number of original articles on mastitis and proteomics by type of work referred to therein (experimental work or field work) and in accord with mammalian species involved in respective studies.

| | Type of work | |
|---|---|---|
| Mammalian species | Experimental work | Field work |
| Buffalo | 0 (0.0%) | 3 (100.0%) |
| Cattle | 17 (22.4%) | 59 (77.6%) |
| Goat | 3 (33.3%) | 6 (66.7%) |
| Sheep | 9 (56.2%) | 7 (43.8%) |
| Rat | 2 (100.0%) | 0 (0.0%) |
| Mouse | 1 (100.0%) | 0 (0.0%) |
| Human | 0 (0.0%) | 1 (100.0%) |
| Camel | 0 (0.0%) | 1 (100.0%) |
| Yak | 0 (0.0%) | 1 (100.0% |

**Figure S4.** Box and whisker plot for year of publication of original articles on mastitis and proteomics by type of work in respective studies: experimental work (blue), field work (green) or laboratory-based work (grey).

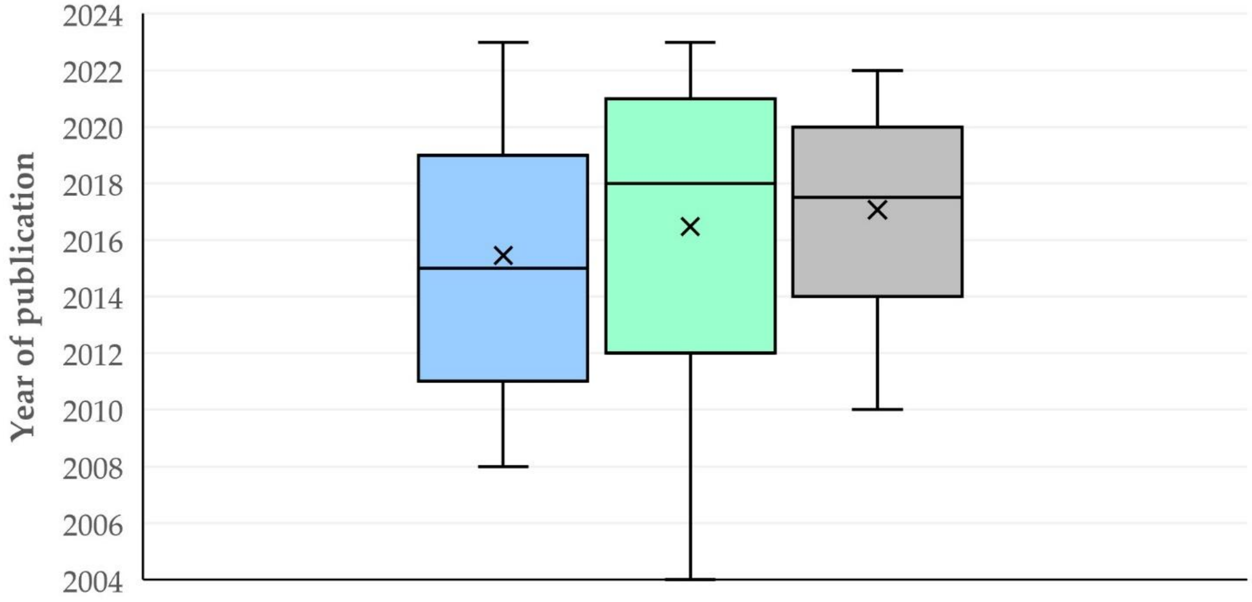

**Figure S5.** Number of original articles on mastitis and proteomics, in accord with material assessed in respective studies.

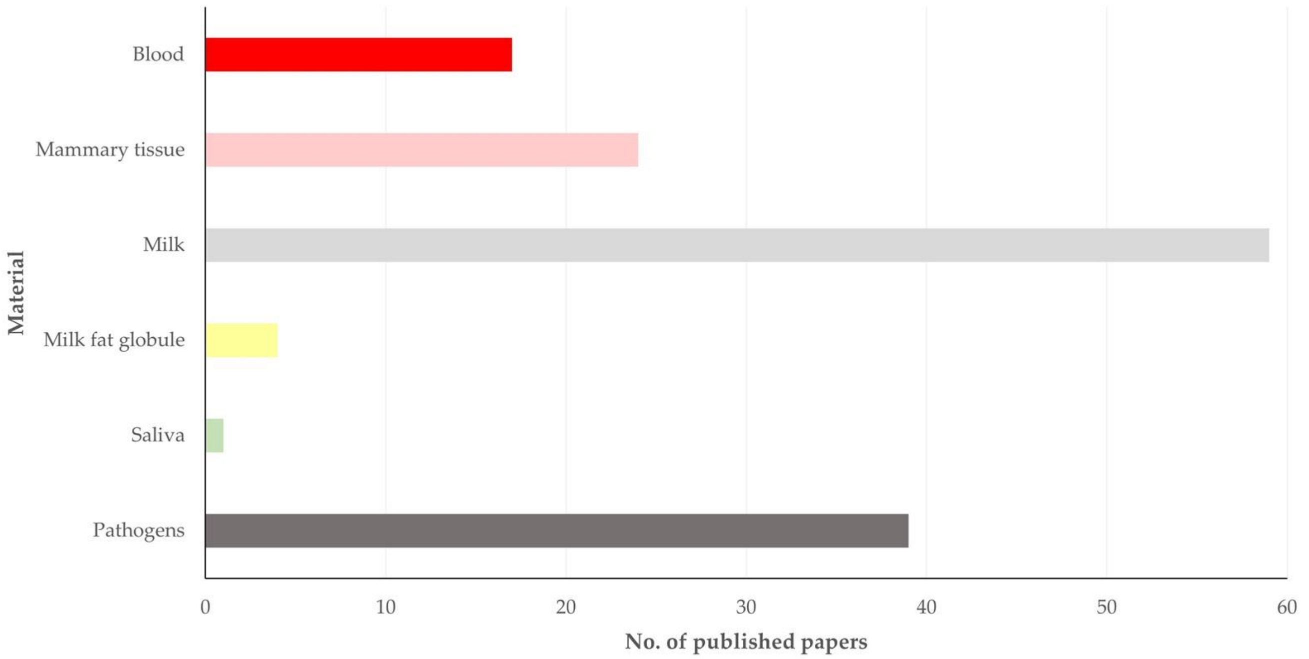

**Table S8.** Number of original articles on mastitis and proteomics, in accord with country of origin and mastitis aspect in respective studies.

| Country [1] | Mastitis aspect | | | |
|---|---|---|---|---|
| | Aetiology | Diagnosis | Pathogenesis | Treatment |
| Brazil | 1 | 4 | 2 | 3 |
| Canada | 0 | 3 | 3 | 0 |
| China | 0 | 15 | 17 | 17 |
| Croatia | 0 | 1 | 3 | 0 |
| Denmark | 0 | 4 | 3 | 0 |
| France | 2 | 0 | 7 | 1 |
| Germany | 0 | 4 | 2 | 0 |
| Greece | 0 | 6 | 1 | 0 |
| Italy | 0 | 2 | 14 | 6 |
| Spain | 0 | 5 | 0 | 2 |
| United Kingdom | 0 | 4 | 4 | 1 |
| United States of America | 0 | 9 | 8 | 2 |

[1] only the 12 countries with most (≥ 5) published papers are included.

**Table S9.** Number of original articles on mastitis and proteomics, in accord with material assessed and mastitis aspect in the respective studies.

| | Material assessed | | | | | |
|---|---|---|---|---|---|---|
| Mastitis aspect studied | Blood | Mammary tissue | Milk | Milk fat globule | Saliva | Pathogens |
| Aetiology | 1 | 0 | 0 | 0 | 0 | 6 |
| Diagnosis | 9 | 9 | 44 | 3 | 0 | 13 |
| Pathogenesis | 11 | 17 | 19 | 1 | 1 | 16 |
| Treatment | 2 | 9 | 5 | 0 | 0 | 13 |

**Table S10.** Pathogens included in studies on mastitis and proteomics and number of original articles describing their evaluation in respective studies.

| Pathogen | No. of articles published |
|---|---|
| *Staphylococcus aureus* | 55 |
| *Escherichia coli* | 31 |
| *Streptococcus uberis* | 19 |
| *Streptococcus agalactiae* | 11 |
| *Staphylococcus chromogenes* | 7 |
| *Mannheimia haemolytica* | 6 |
| *Streptococcus dysgalactiae* | 6 |
| *Staphylococcus epidermidis* | 5 |
| *Staphylococcus xylosus* | 4 |
| *Streptococcus parauberis* | 4 |
| *Prototheca zopfii* | 3 |
| *Staphylococcus canis* | 3 |
| *Staphylococus warneri* | 3 |
| *Streptococcus gallolyticus* | 3 |
| *Corynebacterium bovis* | 2 |
| *Mycoplasma agalactiae* | 2 |
| *Serratia macrescens* | 2 |
| *Staphylococcus auricularis* | 2 |
| *Staphylococcus cohnii* | 2 |
| *Arthrobacter gandavensis* | 1 |
| *Bacillus muralis* | 1 |
| *Bacillus simplex* | 1 |
| *Candida albicans* | 1 |
| *Citrobacter freundii* | 1 |
| coagulase-negative *Staphylococcus* sp. | 1 |
| *Corynebacterium* | 1 |
| *Enterobacter asburieae* | 1 |
| *Enterobacter cloacae* | 1 |
| *Enterococcus faecalis* | 1 |
| *Klebsiella oxytoca* | 1 |
| *Klebsiella pneumoniae* | 1 |
| *Kodameae ohmeri* | 1 |
| *Lactobacillus lactis* | 1 |
| *Mycoplasma bovis* | 1 |
| *Mycoplasma mycoides* | 1 |
| *Paenibacillus odorifer* | 1 |
| *Plasmodium beghei* | 1 |
| *Prototheca blaschkae* | 1 |
| *Staphylococcus capitis* | 1 |
| *Staphylococcus caprae* | 1 |
| *Staphylococcus haemolyticus* | 1 |
| *Staphylococcus hominis* | 1 |
| *Staphylococcus hyicus* | 1 |
| *Staphylococcus lugdunenensis* | 1 |
| *Staphylococcus simulans* | 1 |
| *Streptococcus equinus* | 1 |

**Table S11.** Number of original articles on mastitis associated with *Escherichia coli* or *Staphylococcus aureus* or *Streptococcus uberis* and proteomics, in accord with study details.

| | Bacteria | | |
|---|---|---|---|
| | *Staphylococcus aureus* | *Escherichia coli* | *Streptococcus uberis* |
| Mammalian species | | | |
| Buffalo | 1 | 0 | 1 |
| Cattle | 41 | 25 | 16 |
| Camel | 1 | 0 | 0 |
| Goat | 4 | 3 | 0 |
| Human | 0 | 0 | 0 |
| Mouse | 0 | 0 | 0 |
| Rat | 0 | 0 | 0 |
| Sheep | 8 | 2 | 2 |
| Yak | 1 | 1 | 0 |
| Mastitis aspect | | | |
| Aetiology | 7 | 0 | 1 |
| Diagnosis | 20 | 17 | 8 |
| Pathogenesis | 30 | 12 | 9 |
| Treatment | 14 | 9 | 3 |
| Type of study | | | |
| Field work | 34 | 15 | 10 |
| Experimental work | 11 | 10 | 6 |
| Laboratory-based work | 10 | 6 | 3 |
| Material assessed | | | |
| Blood | 5 | 3 | 2 |
| Mammary tissue | 13 | 7 | 2 |
| Milk | 20 | 18 | 9 |
| Milk fat globule | 1 | 0 | 1 |
| Saliva | 0 | 0 | 1 |
| Pathogens | 17 | 5 | 6 |

**Table S12.** Number of original articles with the various proteomics methodological approaches, in accord with material assessed in respective studies.

| Proteomics methodological approaches [1] | Material assessed | | | | | |
|---|---|---|---|---|---|---|
| | Blood | Mammary tissue | Milk | Milk fat globule | Saliva | Pathogens |
| LC-MS/MS | 5 | 16 | 24 | 2 | 1 | 12 |
| 2-DE, MALDI-TOF MS | 5 | 2 | 12 | 0 | 0 | 8 |
| 2-DE, LC-MS/MS | 5 | 1 | 9 | 0 | 0 | 4 |
| 2D-DIGE, MALDI-TOF MS, GeLC-MS/MS | 1 | 0 | 7 | 2 | 0 | 4 |
| MALDI-TOF MS | 0 | 0 | 2 | 0 | 0 | 8 |
| GeLC-MS/MS | 1 | 0 | 2 | 0 | 0 | 2 |
| Bioinformatics | 0 | 2 | 2 | 0 | 0 | 1 |
| LC-MS/MS, Bioinformatics | 0 | 3 | 0 | 0 | 0 | 0 |
| MALDI-TOF MS, LC-MS/MS | 0 | 0 | 1 | 0 | 0 | 0 |

[1] LC-MS/MS: liquid chromatography-tandem mass spectrometry, 2-DE: two dimensional gel electrophoresis, MALDI-TOF MS: matrix-assisted laser desorption/ionization coupled to time-of-flight mass spectrometry, 2D-DIGE: two-dimensional difference gel electrophoresis, GeLC-MS/MS: polyacrylamide gel electrophoresis followed by liquid chromatography-tandem mass spectrometry.

**Table S13.** Results of pairwise correlation analysis ($r_{sp}$) for proteomics methodological approach, mammalian species, mastitis aspect, material assessed and year of publication described in original articles on mastitis and proteomics.

| | Proteomics approach | Mammalian species | Mastitis aspect | Material assessed | Year of publication |
|---|---|---|---|---|---|
| Proteomics approach | | | | | |
| Mammalian species | 0.19 ($p = 0.009$) | | | | |
| Mastitis aspect | 0.15 ($p = 0.045$) | 0.09 ($p = 0.22$) | | | |
| Material assessed | 0.03 ($p = 0.66$) | 0.12 ($p = 0.10$) | 0.05 ($p = 0.53$) | | |
| Year of publication | 0.40 ($p < 0.0001$) | 0.02 ($p = 0.80$) | 0.15 ($p = 0.046$) | 0.08 ($p = 0.27$) | |

**Table S14.** Eigenvalues for principal component analysis for proteomics approach, mammalian species, mastitis aspect, material assessed and year of publication described in original articles on mastitis and proteomics.

| Parameter | PC1 | PC2 | PC3 | PC4 | PC5 |
|---|---|---|---|---|---|
| Eigenvalue | 1.60 | 1.18 | 0.92 | 0.80 | 0.51 |
| % of Variance | 32.01 | 23.50 | 18.37 | 16.01 | 10.11 |
| Cumulative variance (%) | 32.01 | 55.51 | 73.87 | 89.89 | 100.00 |

**Figure S6.** Scree-plot of results of principal components analysis for proteomics methodological approach, mammalian species, mastitis aspect, material assessed and year of publication in original articles on mastitis and proteomics.

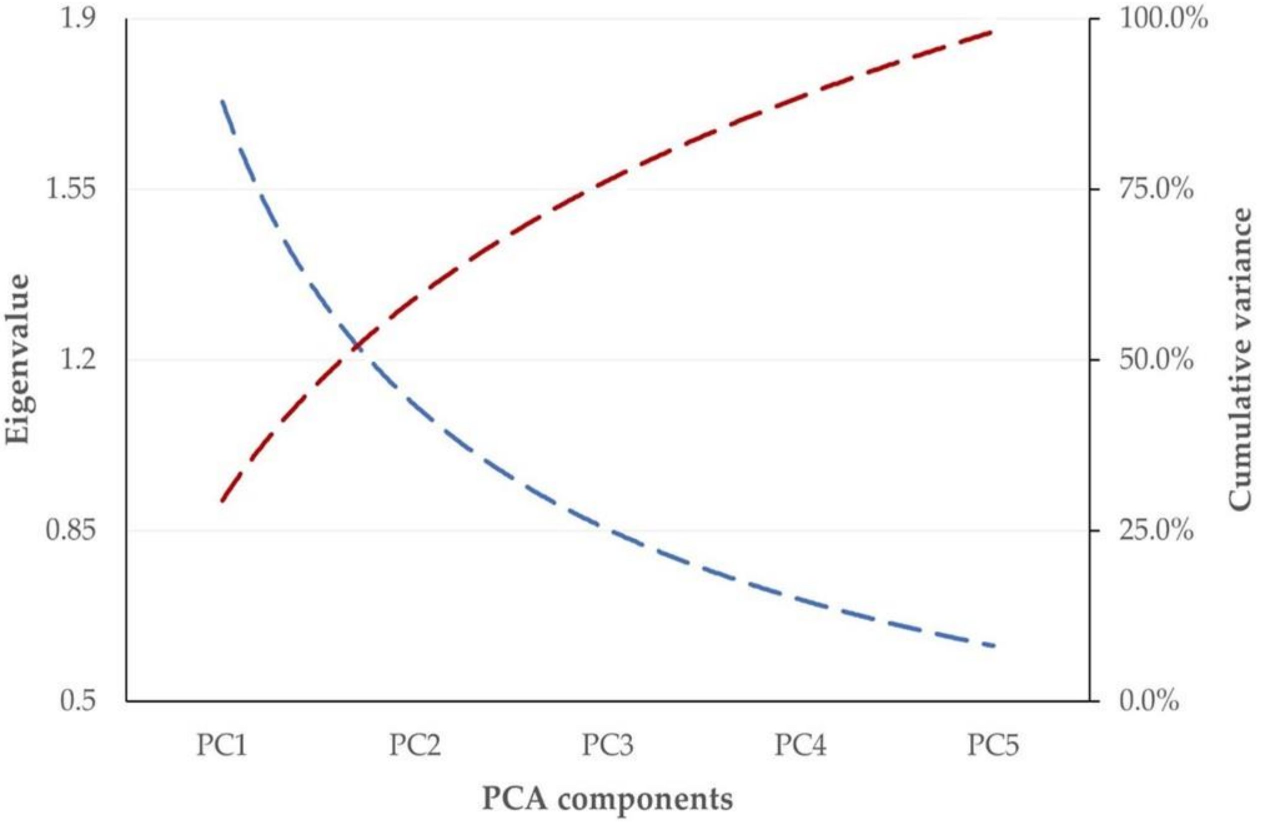

**Figure S7.** Ternary plot of results of principal components analysis for proteomics approach, mammalian species, mastitis aspect, material assessed and year of publication described in papers on mastitis and proteomics.

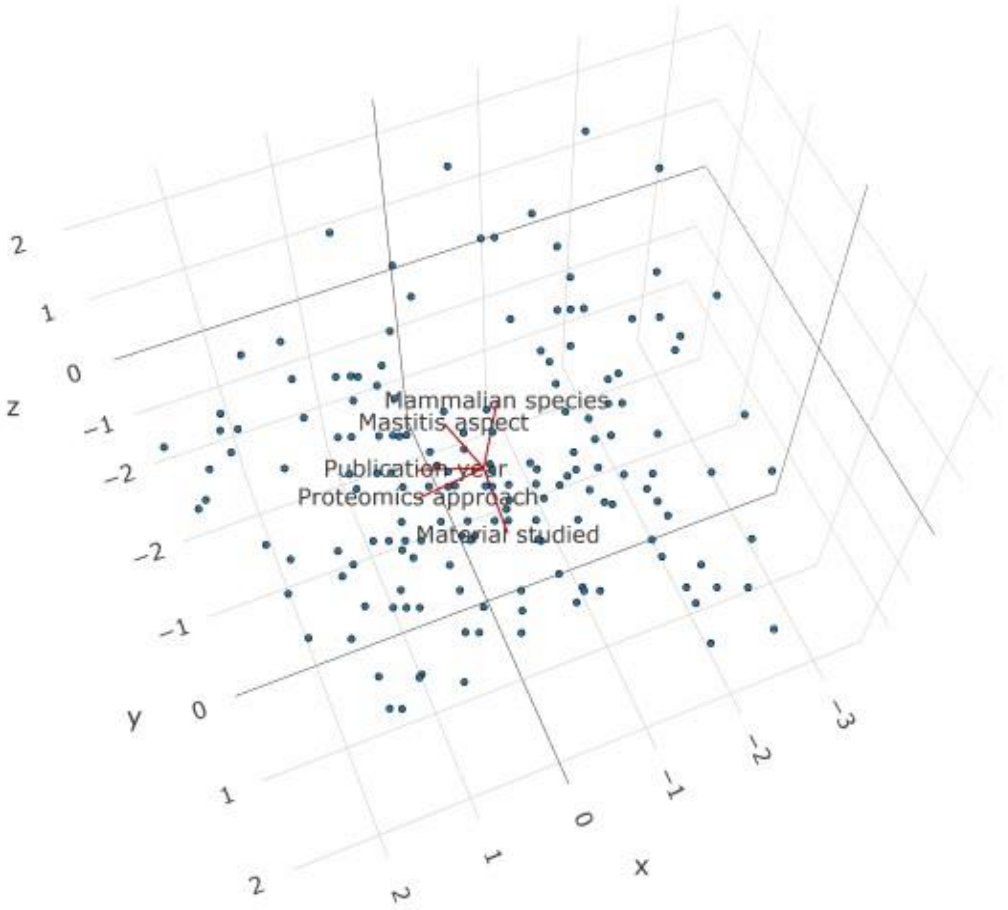

**Table S15.** Heat plot of combinations of proteomics methodological approach, mammalian species, mastitis aspect and year of publication described in original articles on mastitis and proteomics.

| Proteomics methodlogical approach [1] | Year of publication | Mastitis aspect | Mammalian species | No. of articles published |
|---|---|---|---|---|
| 2-DE, MALDI-TOF MS | 2019 | Diagnosis | Sheep | 6 |
| LC-MS/MS | 2020 | Diagnosis | Cattle | 4 |
| LC-MS/MS | 2018 | Pathogenesis | Cattle | 4 |
| LC-MS/MS | 2020 | Pathogenesis | Cattle | 4 |
| LC-MS/MS | 2021 | Pathogenesis | Cattle | 4 |
| LC-MS/MS | 2022 | Treatment | Cattle | 4 |
| 2-DE, MALDI-TOF MS | 2012 | Diagnosis | Cattle | 3 |
| 2D-DIGE, MALDI-TOF MS, GeLC-MS/MS | 2010 | Diagnosis | Cattle | 3 |
| LC-MS/MS | 2018 | Diagnosis | Cattle | 3 |
| 2-DE, LC-MS/MS | 2016 | Diagnosis | Cattle | 3 |
| LC-MS/MS | 2022 | Pathogenesis | Cattle | 3 |
| LC-MS/MS | 2023 | Pathogenesis | Cattle | 3 |
| Bioinformatics | 2022 | Treatment | Cattle | 3 |
| MALDI-TOF MS | 2022 | Aetiology | Cattle | 2 |
| 2-DE, MALDI-TOF MS | 2013 | Diagnosis | Cattle | 2 |
| 2D-DIGE, MALDI-TOF MS, GeLC-MS/MS | 2014 | Diagnosis | Cattle | 2 |
| LC-MS/MS | 2010 | Diagnosis | Cattle | 2 |
| LC-MS/MS | 2016 | Diagnosis | Cattle | 2 |
| LC-MS/MS | 2023 | Diagnosis | Cattle | 2 |
| 2-DE, LC-MS/MS | 2009 | Diagnosis | Cattle | 2 |
| MALDI-TOF MS | 2017 | Diagnosis | Cattle | 2 |
| 2-DE, MALDI-TOF MS | 2013 | Pathogenesis | Cattle | 2 |
| 2D-DIGE, MALDI-TOF MS, GeLC-MS/MS | 2010 | Pathogenesis | Cattle | 2 |
| LC-MS/MS | 2010 | Pathogenesis | Cattle | 2 |
| LC-MS/MS | 2013 | Pathogenesis | Cattle | 2 |
| LC-MS/MS | 2014 | Pathogenesis | Cattle | 2 |
| 2-DE, MALDI-TOF MS | 2016 | Treatment | Cattle | 2 |
| LC-MS/MS | 2018 | Treatment | Cattle | 2 |
| LC-MS/MS | 2019 | Treatment | Cattle | 2 |
| LC-MS/MS | 2020 | Diagnosis | Goat | 2 |
| 2D-DIGE, MALDI-TOF MS, GeLC-MS/MS | 2011 | Diagnosis | Sheep | 2 |
| 2D-DIGE, MALDI-TOF MS, GeLC-MS/MS | 2013 | Diagnosis | Sheep | 2 |
| 2-DE, LC-MS/MS | 2011 | Pathogenesis | Sheep | 2 |
| 2-DE, LC-MS/MS | 2018 | Diagnosis | Buffalo | 1 |
| 2-DE, LC-MS/MS | 2019 | Diagnosis | Buffalo | 1 |
| 2-DE, LC-MS/MS | 2020 | Diagnosis | Buffalo | 1 |
| 2-DE, LC-MS/MS | 2019 | Pathogenesis | Buffalo | 1 |
| MALDI-TOF MS | 2021 | Treatment | Camel | 1 |
| 2-DE, MALDI-TOF MS | 2009 | Aetiology | Cattle | 1 |

| | | | | |
|---|---|---|---|---|
| 2D-DIGE, MALDI-TOF MS, GeLC-MS/MS | 2011 | Aetiology | Cattle | 1 |
| 2-DE, LC-MS/MS | 2022 | Aetiology | Cattle | 1 |
| 2-DE, MALDI-TOF MS | 2004 | Diagnosis | Cattle | 1 |
| 2-DE, MALDI-TOF MS | 2008 | Diagnosis | Cattle | 1 |
| 2-DE, MALDI-TOF MS | 2009 | Diagnosis | Cattle | 1 |
| 2-DE, MALDI-TOF MS | 2011 | Diagnosis | Cattle | 1 |
| 2-DE, MALDI-TOF MS | 2014 | Diagnosis | Cattle | 1 |
| 2-DE, MALDI-TOF MS | 2015 | Diagnosis | Cattle | 1 |
| 2-DE, MALDI-TOF MS | 2018 | Diagnosis | Cattle | 1 |
| 2-DE, MALDI-TOF MS | 2020 | Diagnosis | Cattle | 1 |
| 2D-DIGE, MALDI-TOF MS, GeLC-MS/MS | 2012 | Diagnosis | Cattle | 1 |
| GeLC-MS/MS | 2010 | Diagnosis | Cattle | 1 |
| LC-MS/MS | 2012 | Diagnosis | Cattle | 1 |
| LC-MS/MS | 2013 | Diagnosis | Cattle | 1 |
| LC-MS/MS | 2014 | Diagnosis | Cattle | 1 |
| LC-MS/MS | 2015 | Diagnosis | Cattle | 1 |
| LC-MS/MS | 2017 | Diagnosis | Cattle | 1 |
| LC-MS/MS | 2019 | Diagnosis | Cattle | 1 |
| LC-MS/MS | 2021 | Diagnosis | Cattle | 1 |
| LC-MS/MS | 2022 | Diagnosis | Cattle | 1 |
| 2-DE, LC-MS/MS | 2012 | Diagnosis | Cattle | 1 |
| 2-DE, LC-MS/MS | 2015 | Diagnosis | Cattle | 1 |
| Bioinformatics | 2023 | Diagnosis | Cattle | 1 |
| MALDI-TOF MS | 2012 | Diagnosis | Cattle | 1 |
| MALDI-TOF MS | 2020 | Diagnosis | Cattle | 1 |
| LC-MS/MS, Bioinformatics | 2023 | Diagnosis | Cattle | 1 |
| 2-DE, MALDI-TOF MS | 2004 | Pathogenesis | Cattle | 1 |
| 2-DE, MALDI-TOF MS | 2007 | Pathogenesis | Cattle | 1 |
| 2-DE, MALDI-TOF MS | 2015 | Pathogenesis | Cattle | 1 |
| 2-DE, MALDI-TOF MS | 2020 | Pathogenesis | Cattle | 1 |
| 2D-DIGE, MALDI-TOF MS, GeLC-MS/MS | 2007 | Pathogenesis | Cattle | 1 |
| 2D-DIGE, MALDI-TOF MS, GeLC-MS/MS | 2012 | Pathogenesis | Cattle | 1 |
| GeLC-MS/MS | 2005 | Pathogenesis | Cattle | 1 |
| GeLC-MS/MS | 2010 | Pathogenesis | Cattle | 1 |
| GeLC-MS/MS | 2014 | Pathogenesis | Cattle | 1 |
| LC-MS/MS | 2006 | Pathogenesis | Cattle | 1 |
| LC-MS/MS | 2009 | Pathogenesis | Cattle | 1 |
| LC-MS/MS | 2012 | Pathogenesis | Cattle | 1 |
| LC-MS/MS | 2016 | Pathogenesis | Cattle | 1 |
| LC-MS/MS | 2019 | Pathogenesis | Cattle | 1 |
| 2-DE, LC-MS/MS | 2009 | Pathogenesis | Cattle | 1 |
| 2-DE, LC-MS/MS | 2011 | Pathogenesis | Cattle | 1 |
| 2-DE, LC-MS/MS | 2012 | Pathogenesis | Cattle | 1 |
| 2-DE, LC-MS/MS | 2014 | Pathogenesis | Cattle | 1 |

| | | | | |
|---|---|---|---|---|
| 2-DE, LC-MS/MS | 2020 | Pathogenesis | Cattle | 1 |
| 2-DE, LC-MS/MS | 2022 | Pathogenesis | Cattle | 1 |
| Bioinformatics | 2022 | Pathogenesis | Cattle | 1 |
| MALDI-TOF MS | 2018 | Pathogenesis | Cattle | 1 |
| MALDI-TOF MS | 2021 | Pathogenesis | Cattle | 1 |
| LC-MS/MS, Bioinformatics | 2023 | Pathogenesis | Cattle | 1 |
| 2-DE, MALDI-TOF MS | 2015 | Treatment | Cattle | 1 |
| 2-DE, MALDI-TOF MS | 2020 | Treatment | Cattle | 1 |
| 2D-DIGE, MALDI-TOF MS, GeLC-MS/MS | 2008 | Treatment | Cattle | 1 |
| 2D-DIGE, MALDI-TOF MS, GeLC-MS/MS | 2011 | Treatment | Cattle | 1 |
| LC-MS/MS | 2010 | Treatment | Cattle | 1 |
| LC-MS/MS | 2014 | Treatment | Cattle | 1 |
| LC-MS/MS | 2017 | Treatment | Cattle | 1 |
| LC-MS/MS | 2020 | Treatment | Cattle | 1 |
| Bioinformatics | 2017 | Treatment | Cattle | 1 |
| MALDI-TOF MS | 2018 | Treatment | Cattle | 1 |
| MALDI-TOF MS | 2021 | Treatment | Cattle | 1 |
| LC-MS/MS, Bioinformatics | 2021 | Treatment | Cattle | 1 |
| MALDI-TOF MS | 2021 | Aetiology | Goat | 1 |
| 2D-DIGE, MALDI-TOF MS, GeLC-MS/MS | 2010 | Diagnosis | Goat | 1 |
| GeLC-MS/MS | 2013 | Diagnosis | Goat | 1 |
| 2-DE, LC-MS/MS | 2013 | Diagnosis | Goat | 1 |
| 2-DE, LC-MS/MS | 2015 | Diagnosis | Goat | 1 |
| MALDI-TOF MS, LC-MS/MS | 2020 | Diagnosis | Goat | 1 |
| 2D-DIGE, MALDI-TOF MS, GeLC-MS/MS | 2010 | Pathogenesis | Goat | 1 |
| LC-MS/MS | 2020 | Pathogenesis | Goat | 1 |
| 2-DE, LC-MS/MS | 2013 | Pathogenesis | Goat | 1 |
| LC-MS/MS | 2019 | Treatment | Goat | 1 |
| MALDI-TOF MS | 2021 | Treatment | Goat | 1 |
| LC-MS/MS | 2021 | Pathogenesis | Human | 1 |
| LC-MS/MS, Bioinformatics | 2022 | Pathogenesis | Human | 1 |
| LC-MS/MS | 2021 | Pathogenesis | Mouse | 1 |
| MALDI-TOF MS | 2021 | Treatment | Mouse | 1 |
| LC-MS/MS | 2020 | Pathogenesis | Rat | 1 |
| LC-MS/MS | 2022 | Treatment | Rat | 1 |
| 2-DE, MALDI-TOF MS | 2009 | Aetiology | Sheep | 1 |
| 2-DE, MALDI-TOF MS | 2013 | Diagnosis | Sheep | 1 |
| 2-DE, MALDI-TOF MS | 2020 | Diagnosis | Sheep | 1 |
| 2-DE, MALDI-TOF MS | 2021 | Diagnosis | Sheep | 1 |
| LC-MS/MS | 2015 | Diagnosis | Sheep | 1 |
| Bioinformatics | 2023 | Diagnosis | Sheep | 1 |

| | | | | |
|---|---|---|---|---|
| 2-DE, MALDI-TOF MS | 2009 | Pathogenesis | Sheep | 1 |
| 2-DE, MALDI-TOF MS | 2019 | Pathogenesis | Sheep | 1 |
| GeLC-MS/MS | 2013 | Pathogenesis | Sheep | 1 |
| LC-MS/MS | 2022 | Pathogenesis | Sheep | 1 |
| 2-DE, LC-MS/MS | 2012 | Pathogenesis | Sheep | 1 |
| 2-DE, LC-MS/MS | 2011 | Treatment | Sheep | 1 |
| 2-DE, MALDI-TOF MS | 2014 | Diagnosis | Yak | 1 |
| 2-DE, MALDI-TOF MS | 2014 | Treatment | Yak | 1 |

[1] LC-MS/MS: liquid chromatography-tandem mass spectrometry, 2-DE: two dimensional gel electrophoresis, MALDI-TOF MS: matrix-assisted laser desorption/ionization coupled to time-of-flight mass spectrometry, 2D-DIGE: two-dimensional difference gel electrophoresis, GeLC-MS/MS: polyacrylamide gel electrophoresis followed by liquid chromatography-tandem mass spectrometry.

**Table S16.** Original articles on mastitis and proteomics, describing involvement of additional -omics technologies, in accord with material assessed in respective studies.

| Material assessed in studies | Involvement of additional -omics technologies | No involvement of additional -omics technologies in paper |
|---|---|---|
| Blood | 1 | 16 |
| Mammary tissue | 5 | 19 |
| Milk | 6 | 53 |
| Milk fat globule | 0 | 4 |
| Saliva | 0 | 1 |
| Pathogens | 13 | 26 |

**Table S17.** Journals in which papers on mastitis and proteomics were published and respective number of papers.

| Journals | No. of published papers |
|---|---|
| *Journal of Proteomics* | 16 |
| *Journal of Dairy Science* | 12 |
| *Journal of Proteome Research* | 8 |
| *International Journal of Molecular Sciences* | 6 |
| *Veterinary Microbiology* | 6 |
| *Animals* | 5 |
| *Data in Brief* | 5 |
| *Veterinary Research* | 5 |
| *Proteomics* | 4 |
| *Animal* | 3 |
| *Frontiers in Microbiology* | 3 |
| *Journal of Agricultural and Food Chemistry* | 3 |
| *PloS One* | 3 |
| *Research in Veterinary Science* | 3 |
| *Scientific Reports* | 3 |
| *Veterinary Immunology and Immunopathology* | 3 |
| *BMC Veterinary Research* | 2 |
| *Frontiers in Veterinary Science* | 2 |
| *Infection and Immunity* | 2 |
| *Journal of Dairy Research* | 2 |
| *Journal of Mammary Gland Biology and Neoplasia* | 2 |
| *Pathogens* | 2 |
| *Agricultural Sciences in China* | 1 |
| *Analytical and Bioanalytical Chemistry* | 1 |
| *Animal Biotechnology* | 1 |
| *Animal Production Science* | 1 |
| *Animal Science Journal* | 1 |
| *Antibiotics* | 1 |
| *Antioxidants* | 1 |
| *Applied and Environmental Microbiology* | 1 |
| *Archiv fur Tierzucht* | 1 |
| *Archives of Microbiology* | 1 |
| *Arquivo Brasileiro de Medicina Veterinaria e Zootecnia* | 1 |
| *Biological Trace Element Research* | 1 |
| *Biology* | 1 |
| *BMC Genomics* | 1 |
| *BMC Microbiology* | 1 |
| *Brazilian Journal of Microbiology* | 1 |
| *Bulletin of the Veterinary Institute in Pulawy* | 1 |
| *Carbohydrate Polymers* | 1 |
| *Cell Stress & Chaperones* | 1 |
| *Clinical Proteomics* | 1 |
| *Colloids and Surfaces B-Biointerfaces* | 1 |
| *Current Microbiology* | 1 |
| *Current Proteomics* | 1 |
| *Czech Journal of Animal Science* | 1 |
| *Food Research International* | 1 |

| | |
|---|---|
| *Foods* | 1 |
| *Frontiers in Animal Science* | 1 |
| *Frontiers in Immunology* | 1 |
| *Frontiers in Pharmacology* | 1 |
| *Genetics and Molecular Research* | 1 |
| *International Dairy Journal* | 1 |
| *International Immunopharmacology* | 1 |
| *International Journal of Dairy Technology* | 1 |
| *Journal of Microbiological Methods* | 1 |
| *Journal of Molecular Biology* | 1 |
| *Journal of Pharmacy and Pharmacology* | 1 |
| *Journal of Veterinary Diagnostic Investigation* | 1 |
| *Journal of Veterinary Science* | 1 |
| *Malaysian Journal of Microbiology* | 1 |
| *Medical Mycology* | 1 |
| *Medical Oncology* | 1 |
| *Microbial Ecology* | 1 |
| *Microbial Pathogenesis* | 1 |
| *Microorganisms* | 1 |
| *Molecular Biosystems* | 1 |
| *PeerJ* | 1 |
| *Pesquisa Veterinaria Brasileira* | 1 |
| *Proteome Science* | 1 |
| *Tropical Animal Health and Production* | 1 |
| *Tropical Biomedicine* | 1 |
| *Vaccine* | 1 |
| *Veterinary Journal* | 1 |
| *Veterinary Research Communications* | 1 |
| *Veterinary World* | 1 |
| *Virulence* | 1 |
| *Virus Genes* | 1 |

**Table S18.** Association between journals and countries of origin of published papers on mastitis and proteomics.

| | Journal [2] | | | | | | | |
|---|---|---|---|---|---|---|---|---|
| Country [1] | *Animals* | *Data Brief* | *Int. J. Mol Sci.* | *J. Dairy Sci.* | *J. Proteome Res.* | *J. Proteom.* | *Vet. Microbiol.* | *Vet. Res.* |
| Brazil | 0 | 0 | 0 | 3 | 0 | 0 | 0 | 0 |
| Canada | 0 | 1 | 1 | 1 | 1 | 0 | 0 | 0 |
| China | 3 | 0 | 2 | 0 | 0 | 0 | 0 | 1 |
| Croatia | 1 | 0 | 0 | 0 | 0 | 2 | 0 | 0 |
| Denmark | 0 | 0 | 0 | 2 | 1 | 1 | 0 | 0 |
| France | 0 | 0 | 1 | 0 | 0 | 1 | 2 | 2 |
| Germany | 0 | 0 | 1 | 0 | 0 | 1 | 0 | 0 |
| Greece | 1 | 2 | 1 | 0 | 0 | 1 | 1 | 0 |
| Italy | 0 | 1 | 0 | 0 | 0 | 6 | 2 | 1 |
| Spain | 0 | 0 | 0 | 0 | 0 | 1 | 0 | 0 |
| United Kingdom | 0 | 0 | 0 | 0 | 1 | 2 | 0 | 0 |
| United States of America | 0 | 1 | 1 | 5 | 2 | 3 | 0 | 0 |

[1] only the 12 countries with most (≥ 5) published papers are included.
[2]: abbreviations of journals from left to right: *Animals*, *Data in Brief*, *International Journal of Molecular Sciences*, *Journal of Dairy Science*, *Journal of Proteome Research*, *Journal of Proteomics*, *Veterinary Microbiology*, *Veterinary Research*.

**Table S19.** Sub-categories of journals in Web-of Science, in which papers on mastitis and proteomics were published, and respective number of papers.

| Sub-categories of journals | No. of published papers |
|---|---|
| Veterinary Sciences | 39 |
| Biochemical Research Methods | 33 |
| Agriculture, Dairy & Animal Science | 26 |
| Microbiology | 22 |
| Food Science & Technology | 20 |
| Multidisciplinary Sciences | 12 |
| Biochemistry & Molecular Biology | 11 |
| Immunology | 10 |
| Infectious Diseases | 5 |
| Chemistry, Multidisciplinary | 6 |
| Agriculture, Multidisciplinary | 4 |
| Pharmacology & Pharmacy | 4 |
| Chemistry, Applied | 4 |
| Biotechnology & Applied Microbiology | 3 |
| Endocrinology & Metabolism | 3 |
| Genetics & Heredity | 3 |
| Oncology | 3 |
| Biology | 1 |
| Biophysics | 1 |
| Cell Biology | 1 |
| Chemistry, Analytical | 1 |
| Chemistry, Medicinal | 1 |
| Chemistry, Organic | 1 |
| Chemistry, Physical | 1 |
| Ecology | 1 |
| Marine & Freshwater Biology | 1 |
| Materials Science, Biomaterials | 1 |
| Medicine, Research & Experimental | 1 |
| Mycology | 1 |
| Parasitology | 1 |
| Physiology | 1 |
| Polymer Science | 1 |
| Tropical Medicine | 1 |
| Virology | 1 |

**Figure S8.** Venn diagrams of three pairs of authors, affiliated with scientific establishments in three differ-ent countries, with number of published papers in which they were first or last authors (descriptors of authors not corresponding to their names).

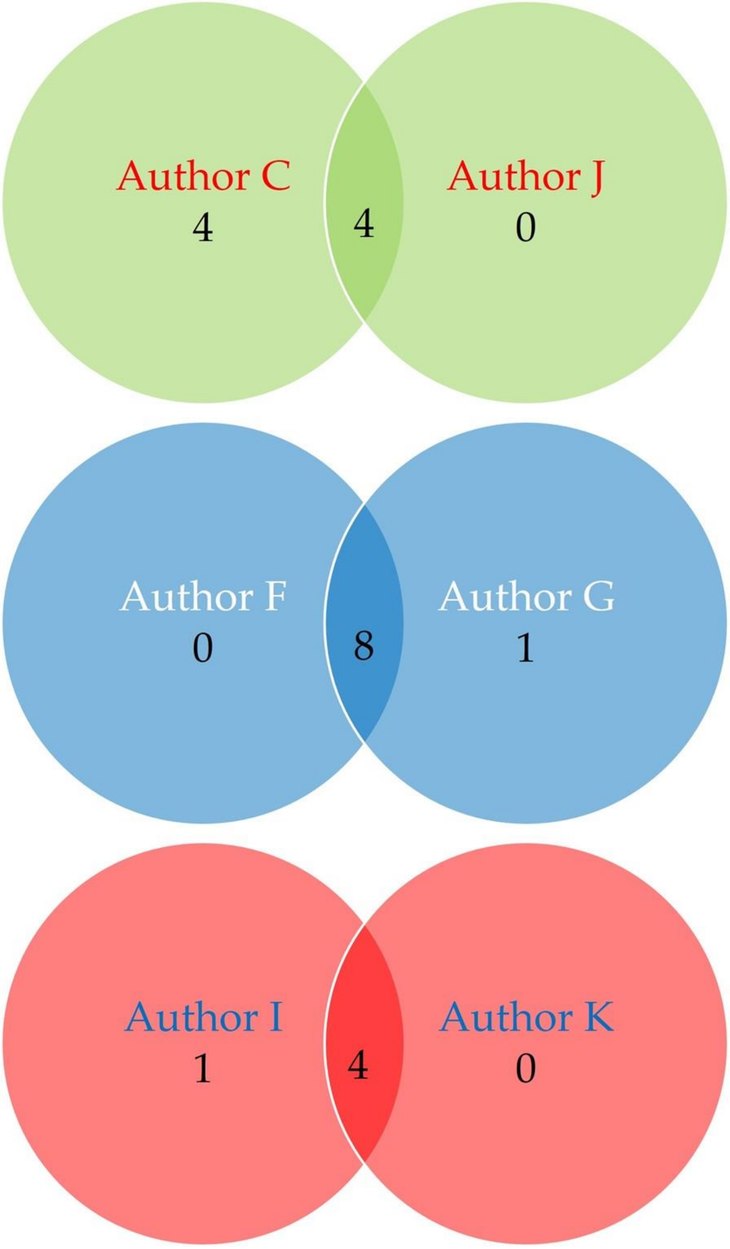

**Table S20.** Median (interquartile range) number of authors per published paper on mastitis and proteomics, in accord with countries of origin of the papers.

| Country | Median (interquartile range) number of authors in published papers |
|---|---|
| Brazil | 8 (1.5) |
| Canada | 4.5 (1.8) |
| China | 8 (4) |
| Croatia | 12 (4) |
| Denmark | 6 (1) |
| France | 9 (8) |
| Germany | 4.5 (2.5) |
| Greece | 8.5 (3.3) |
| Italy | 9 (3) |
| Spain | 8 (2) |
| United Kingdom | 6 (3) |
| United States of America | 4 (4.3) |

**Table S21.** Median (interquartile range) number of yearly citations received by published papers on mastitis and proteomics, in accord with countries of origin of papers.

| Country | Median (interquartile range) number of citations received by published papers |
|---|---|
| Argentina | 0.8 (0.0) |
| Australia | 8.3 (0.0) |
| Brazil | 0.7 (1.1) |
| Canada | 2.9 (2.0) |
| China | 1.6 (1.6) |
| Colombia | 0.3 (0.3) |
| Croatia | 6.8 (7.1) |
| Czech Republic | 4.5 (0.0) |
| Denmark | 4.0 (1.6) |
| Egypt | 1.8 (0.0) |
| Finland | 1.6 (0.0) |
| France | 2.6 (2.0) |
| Germany | 2.2 (2.0) |
| Greece | 0.7 (1.7) |
| Hungary | 0.5 (0.0) |
| India | 3.7 (1.0) |
| Ireland | 2.2 (0.3) |
| Italy | 2.8 (2.5) |
| Japan | 1.2 (0.0) |
| Korea | 1.1 (0.0) |
| New Zealand | 3.1 (3.8) |
| Norway | 0.0 (0.0) |
| Pakistan | 9.3 (0.0) |
| Poland | 2.7 (0.0) |
| Portugal | 5.3 (3.0) |
| Russia | 3.3 (0.0) |
| Saudi Arabia | 1.2 (0.0) |
| Spain | 2.6 (2.2) |
| Thailand | 1.3 (0.0) |
| The Netherlands | 2.5 (0.3) |
| Turkey | 1.3 (0.0) |
| United Kingdom | 2.8 (4.4) |
| United States of America | 2.7 (1.2) |

**Table S22.** Statistical significance of evaluation for potential association of variables of paper content with yearly number of citations.

| Paper details | *p* |
|---|---|
| Mammalian species | 0.39 |
| Mastitis aspect | 0.22 |
| Type of work | 0.14 |
| Material assessed | 0.23 |
| Proteomics technologies employed | 0.44 |
| Additional -omics technologies described | 0.08 |
